# Hand-portable HPLC with broadband spectral detection enables analysis of complex polycyclic aromatic hydrocarbon mixtures

**DOI:** 10.1038/s42004-021-00457-7

**Published:** 2021-02-16

**Authors:** Stelios Chatzimichail, Faraz Rahimi, Aliyah Saifuddin, Andrew J. Surman, Simon D. Taylor-Robinson, Ali Salehi-Reyhani

**Affiliations:** 1 Department of Surgery and Cancer, Imperial College London, London, W12 0HS UK; 2 Department of Chemistry, King’s College London, London, SE1 1DB UK; 3 Institute of Molecular Sciences & Engineering, Imperial College London, London, SW7 2AZ UK

**Keywords:** Microfluidics, Cheminformatics, Environmental monitoring

## Abstract

Polycyclic aromatic hydrocarbons (PAHs) are considered priority hazardous substances due to their carcinogenic activity and risk to public health. Strict regulations are in place limiting their release into the environment, but enforcement is hampered by a lack of adequate field-testing procedure, instead relying on sending samples to centralised analytical facilities. Reliably monitoring levels of PAHs in the field is a challenge, owing to the lack of field-deployable analytical methods able to separate, identify, and quantify the complex mixtures in which PAHs are typically observed. Here, we report the development of a hand-portable system based on high-performance liquid chromatography incorporating a spectrally wide absorption detector, capable of fingerprinting PAHs based on their characteristic spectral absorption profiles: identifying 100% of the 24 PAHs tested, including full coverage of the United States Environmental Protection Agency priority pollutant list. We report unsupervised methods to exploit these new capabilities for feature detection and identification, robust enough to detect and classify co-eluting and hidden peaks. Identification is fully independent of their characteristic retention times, mitigating matrix effects which can preclude reliable determination of these analytes in challenging samples. We anticipate the platform to enable more sophisticated analytical measurements, supporting real-time decision making in the field.

## Abstract (web summary)

Portable liquid chromatography instruments enable a myriad of applications in field research. Here a handportable system incorporating a broadband absorption detector is used to separate and classify polycyclic aromatic hydrocarbons from environmental water samples based solely on spectral fingerprinting.

## Introduction

Polycyclic aromatic hydrocarbons (PAHs) are a class of persistent organic pollutants, of concern since many are variously carcinogenic, mutagenic, or teratogenic. They are ubiquitous on earth, and beyond: their abundance in the interstellar medium has sparked great interest in their formation and their potential role in abiogenesis^1–3^, while on the modern earth they are found naturally in fossil fuels, and formed from both natural or anthropogenic combustion sources^4^. High incidence of cancer due to the occupational exposure to PAHs in the 18th and 19th centuries played a historic role in the understanding of chemical carcinogenesis; scrotal cancers in chimney sweeps and skin cancers in coal tar industry workers being notable examples. Work in the early 20th century culminated in the association the carcinogenic activity of soots and tars to PAHs, particularly benzo[a]pyrene^5^. A broad range of diseases have been associated with exposure to PAHS, including various cancers, as well as metabolic and cardiovascular disease^6–10^. Furthermore, *in utero* and childhood exposure to PAHs has been associated with respiratory defects, cognitive impairment, and low birthweight^11,12^. General population exposure to PAHs can occur via polluted air, contaminated drinking water, and food^13–18^. Due to their effects on health, the ability to accumulate in biota and food chains, they are considered priority hazardous substances and so there exist strict regulations of PAHs by governmental food and environmental agencies around the world.

**Fig. 1:**
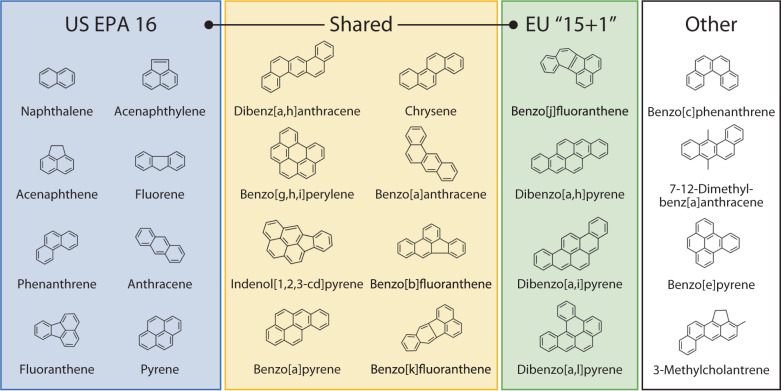
PAHs are structurally diverse and chemically similar. Structures of all 24 PAHs addressed in this study, which include all those identified by EU and US authorities as of particular concerns. US EPA 16 (blue and yellow boxes): All 16 PAHs listed in the United States Environmental Protection Agency priority pollutants list. EU “15 + 1”: European Union priority PAHs (green and yellow boxes). Note, 12 PAHs listed in the EU “15 + 1” are tested here.

PAHs are uncharged, non-polar organic molecules composed of multiple fused aromatic rings. Many PAHs are known (see Fig. 1 and Supplementary Table 1): unsubstituted hydrocarbons, their chemistry, and chromatographic behaviour is similar. In our environment, PAHs are typically observed as complex mixtures with many similar species, making their separation, identification, and quantification particularly challenging. For example, most environmental authorities consider many different PAHs to be of concern (e.g., United States Environmental Protection Agency includes 16 in their priority pollutant list, and some of which are very similar (isomers with the same molecular formula), presenting a considerable analytical challenge. Higher molecular weight PAHs are generally insoluble in water and adsorb on to fine-grained, organic-rich sediments, such as air particulates and soils. While this limits the environmental mobility of higher molecular weight PAHs, they are less accessible for degradation and thereby increase their persistence and (bio)accumulation^19,20^. Lower molecular weight PAHs have relatively higher solubilities in water and tend to be dispersed as contaminants in water sources, making them more available for biological uptake and accumulation in the food chain^21^. Analysis for PAHs in water (environmental, potable) is, therefore, a priority, and an analytical challenge.

Lab-based analytical methods have been established for the determination of PAHs using high performance liquid chromatography (HPLC) and gas chromatography with mass spectrometric detection (GC-MS)^22,23^. Samples, such as water, soil, or sludge, are obtained on-site and subsequently processed and analysed at a centralised facility or laboratory^24,25^. These lab-bound methods are valuable, but there is a growing demand for on-site/‘field’ analysis. The deployment of analytical systems in the field is not trivial, owing to their size/weight and reliance on lab infrastructure (mains electric power, gas supply, solvent supply, waste disposal, etc). Over the last decade, a number of prototype portable analytical systems based on GC, LC, or MS have been reported:^26–29^ most portable systems only partially overcome the challenge, compromising either analytical performance or portability. Portable GC-MS systems using small ion traps have been reported with commercial systems based on these systems being available^30,31^. It is challenging to achieve an optimum method using a single analytical technique, which for PAH analysis has led to the prominent use of both LC and GC based approaches^32–35^. GC-MS methods are able to identify PAHs based on their mass but can be limited to the analysis of those PAHs which are volatile and thermally stable^36,37^. On the other hand, some LC based methods have the advantage of being able to measure PAH isomers that are not easily quantified by GC-MS, yet have been reported to be more prone to matrix interference^32^. Portable LC has shown particular promise as a field-deployable platform for simple analytical tasks (see ref. ^30^ for a treatment of some of these challenges), as it requires neither high vacuum nor gaseous mobile phase^38^. However, so far only instruments with single wavelength UV–vis absorption detectors or combinations of single-wavelength sources, have been reported, with methods relying on retention time alone to identify analytes^39–41^. This is limiting when analysing complex mixtures, where many species elute with minimal resolution, since matrix effects can unpredictably alter characteristic retention times (even leading to-co-elution)^42,43^. In lab-based analysis this is overcome by sample preparation protocols (reducing matrix effects) and hyphenation with MS (adding an extra dimension, *m/z*, to output data to overcome the limited discriminating power of single-wavelength UV detectors). Neither approach is practical in the field; elaborate sample preparation is not feasible and no genuinely portable LC-MS system has yet been reported. Therefore, a new approach to field-based analysis of complex mixtures is sorely needed.

Herein we present efforts to develop a field-based approach to analyse a wide range of environmentally-important PAHs, independent of centralised/lab-based facilities. We report the development of a new portable HPLC instrument with a UV–vis spectral detector, providing an extra dimension to output data: portable enough to carry in a rucksack, with chromatographic performance and stability which compare favourably with a typical lab-based commercial instrument. We also demonstrate that the new capabilities offered by this instrument (3D time-resolved spectral data, rather than 2D single-wavelength absorbance data) may be exploited to analyse a chromatographically challenging mixture of PAHs. Analytes are identified based on their distinctive absorption spectra using a ‘fingerprinting’ approach which is independent of retention times (references sourced from our own measurements, and publicly available databases)^44,45^. We also showed that the 3D data provided by our system allowed us to detect and characterise features that would otherwise remain hidden, using spectral deconvolution and chemometric approaches.

Not only is our platform and methodology of relevance to efforts to monitor PAHs, it is versatile and adaptable to study other complex mixtures in the field. Fingerprinting does not necessarily require the acquisition and curation of reference data, but can rely on publicly available spectral databases to enable positive identification.

## Results

### Development of portable LC system with UV–vis spectral detector

**Fig. 2:**
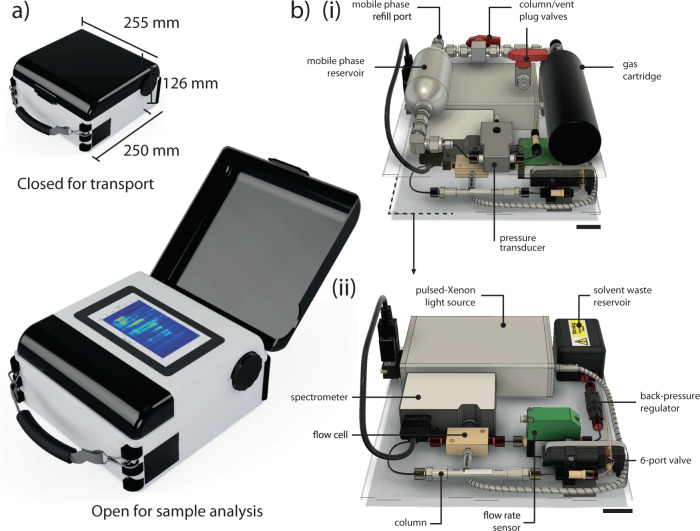
The *anywhereHPLC* portable chromatography system. **a** External view of the case, indicating the dimensions in transport. A protective cover is opened for sample analysis. The system may be operated stationary or in transit. **b** A view of the system’s internal components. (i) The top layer houses the pumping system. (ii) The bottom layer houses the downstream fluidics, sensors, and broadband absorption detector. Black scale bars 25 mm.

We report a stand-alone hand-portable LC system that incorporates all chromatographic componentry and computational units into a single portable system (Fig. 2). While it may be operated in the laboratory, the system is primarily designed to be rugged for operation in challenging field environments and is capable of continuous operation during mechanical shocks, such as 1 m vertical drop tests^26^. It weighs 4.2 kg fully charged with a 150 mL mobile phase and is transported using a handle or shoulder strap. The constant pressure pumping system is capable of driving pressures up to 300 bar and exploits the stored energy of pre-compressed gas, which does not require electrical power to operate in-situ. Since the only drain by the ‘zero-electrical power’ pump is the micro-valve system for switching, the main sources of battery drain are the UV–vis absorption detection system and electronics, which permits continuous operation in excess of 24 h on battery (10 Ah). For the work presented here, the mobile phase reservoir supports up to 19.1 h of continuous operation, which can be extended with the use of larger reservoirs or recharging. Using single-board microcontrollers, optimised for low power consumption, can extend the operating time further. The electrical power consumption of the system is sufficiently low to be supported by USB-attached solar panels, thereby limiting the operation of the device to the volume of solvent, carried on board and in recharging containers. A unique advantage of miniaturised, portable LC systems is their low solvent consumption, particularly when driving micro-bore and capillary columns.

Of course, even the low volumes reported here and elsewhere would not be permitted to be discarded in the field, but the system does ensure longevity of operation. An interesting proposal, in this regard, would be the work of Welch and colleagues, who explored the use of commonly available spirits as green solvent replacements that can be disposed of in ordinary waste or recycling streams for green chromatography^46^. The protective cover is closed during transport and opened for sample injection (Fig. 2a). Samples may be injected directly by cartridge or syringe filling a fixed volume 5 µL sample injection loop, or interfaced with an autosampler (1290 Infinity II Multisampler; Agilent, UK) for autonomous operation. The system is user-controlled via a touch screen panel, which displays chromatographic data (1D and 2D) and on-board sensor data i.e., system pressure, mobile-phase flow rate, and temperature. Access ports permit the facile recharging or switching of the gas cartridge, solvent reservoir, and column. Solvent waste is safely discharged into a sealed on-board reservoir, which may be swapped or drained in the field (Supplementary Fig. 1). The system is also capable of being interfaced with downstream analysers, such as mass spectrometers for hyphenated LC.

**Fig. 3:**
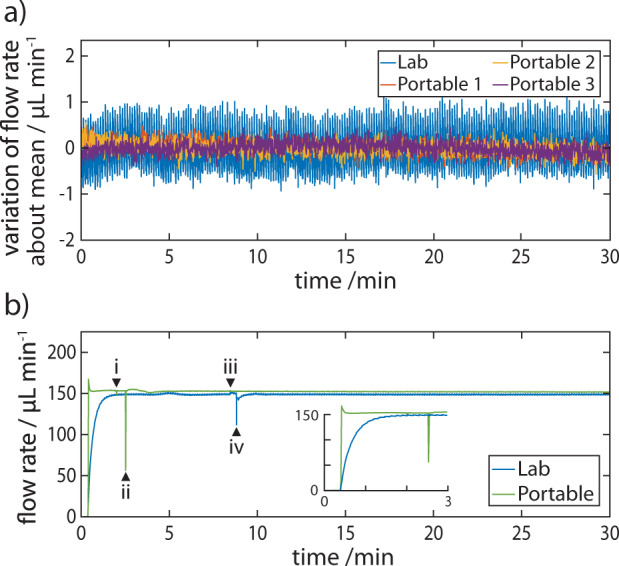
Flow Rate Stability. **a** The variation in flow rate is measured when the nominal flow rate is established for the portable (‘Portable’) and compared to that of a commercial HPLC (‘Lab’ - Agilent 1260/90 system; Agilent, UK). **b** Profile of the change in flow rate when the pump is switched on and during a chromatographic run. The black arrowheads indicate valves switching to (i) loading and (ii) injection on the portable (‘Portable’, green line) and (iii) loading and (iv) injection on the Agilent system (blue line).

A critical component of portable LC is the pump and its performance. We have previously shown the capability of the pump in driving capillary-based chromatographic separations, noting the responsiveness of flow rate to changes in pressure^26^. Here, we assess the stability of flow rate in comparison to a commercial HPLC, driven by a quaternary pump (1260 Infinity II Quaternary Pump; Agilent, UK) (Fig. 3). We measured the mobile phase flow rate on both systems using a solid state in-line flow meter (SLI-0430; Sensirion, Switzerland) at a sampling rate of 1 Hz, positioned downstream of the flow cell and upstream of a 75 psi backpressure regulator, discharging to waste. Mobile phase flow rate may be controlled by altering gas pressure in the pump system, controlled using an output regulator on the gas cartridge. The stability of the flow rate over time was determined by calculating the absolute variation of the flow rate about the mean (Fig. 3a). The portable gas pump exhibited significantly lower *variation* in flow rate; 0.097% RSD (152.2 ± 0.15 µL min^−1^) for the portable and 0.29% RSD (148.8 ± 0.43 µL min^−1^) for the commercial quaternary pump. Whereas, the commercial pump exhibited a significantly higher precision in absolute flow rate; 0.089 µL min^−1^ standard deviation for the commercial quaternary pump and 0.22 µL min^−1^ standard deviation for the portable machine. For long-term pump performance see Supplementary Fig. 2. In addition to flow rate stability, we monitored the response of each system from ‘off’ to ‘on’ (Fig. 3b). We note the responsiveness of the portable system, being able to achieve its intended flow rate within 15 s compared to >90 s for the quaternary pump (Fig. 3b inset).

### Broadband spectral absorption detector evaluation

The identification of species using LC-MS is not easily translated to the field. Instead, spectroscopic fingerprinting is desirable to help ameliorate the limited specificity of LC. It is worth noting that there are challenges to detecting isomeric PAHs using MS-based detection, owing to many analytes possessing identical masses. HPLC absorption detectors typically have a broad wavelength range spanning the deep UV range and much of the visible portion of the spectrum, with spectral discrimination using variable wavelength detectors (VWD) or diode array detectors (DAD) becoming increasingly commonplace in the laboratory. Portable HPLC poses a number of demanding challenges to instrument design and development. Ultimately, systems must be lightweight and be battery operable for extended periods of time. However, conventional lamp-based detectors are heavy and consume in the order of 100 W of power. For example, a modern VWD and DAD consume 85 W and 130 W of electrical power, respectively (Agilent 1260 series). This has led to the ubiquitous use of light-emitting diodes (LEDs) in portable systems requiring absorption detection^38^. This is due to their small size, relatively low power consumption, and potentially favourable performance, in terms of noise and drift, in these systems. LEDs are narrowband emitters; in that they have a narrow spectral output of about a maximum. For defined assays that require only a single absorption wavelength for detection, LEDs are adequate for portable systems. For mixtures containing analytes with a range of chromophores, multiple LEDs may be combined to help mitigate their spectral limitations. One approach would be to install multiple flow cells in series and detect at multiple absorption wavelengths. The use of multiple flow cells is not desirable due to analyte dispersion and cost. This is less of an issue with on-column detection strategies, which can minimise the volume between detectors^40^. LEDs may, instead, be coupled using fibre optics and their output combined at a single flow cell^41^. This may be achieved using fibre-optic bundles, which are assemblies that contain multiple fibres in a single cable. A common implementation is the bifurcated assembly to combine light from 2 sources to extend the spectral range of the measurement. Multi-furcated assemblies combining over 8 sources are commercially available. Achieving a broad spectral range by combining LEDs is possible but may be impractical for a field instrument.

**Fig. 4:**
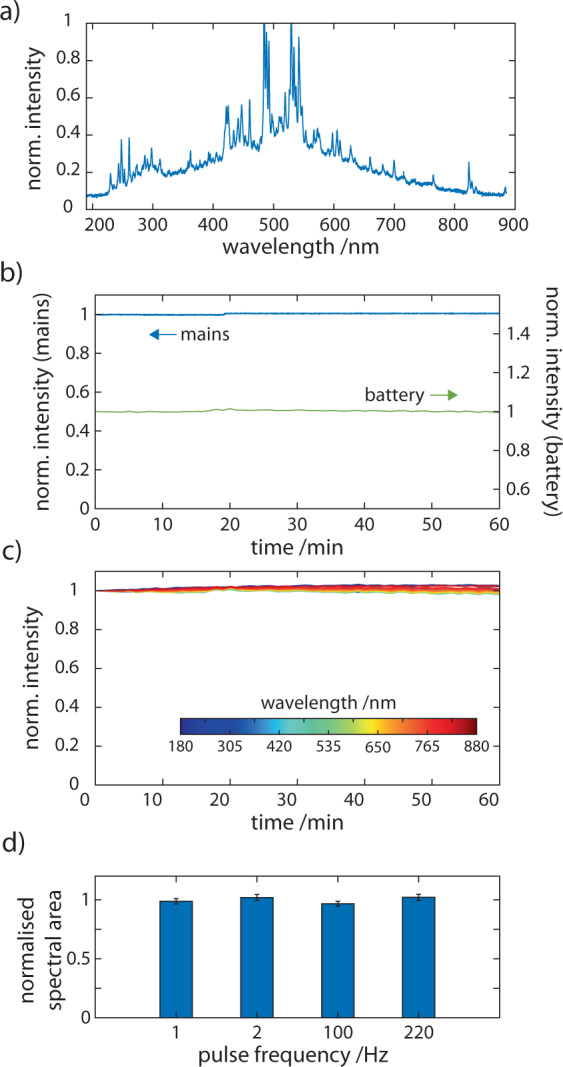
PX light source for portable chromatography. **a** The spectral emission profile of the light source used in this study showing emission from 180 to 890 nm. **b**) To test stability, total intensity is measured overtime of the PX light source measured by a spectrometer when powered by mains power and by battery. Intensity is normalised to that at t = 0 min. **c** Shown here is the variation of intensity at wavelengths across the spectral range. **d** The flow cell was filled with the 24 component PAH mix and irradiated for 30 min at the given pulse frequency (*n* = 3). Sample degradation was quantified using the total area under the absorbance curve.

To overcome these limitations, we used a compact pulsed Xenon (PX) light source coupled via a flow cell to a solid-state spectrometer to achieve a miniaturised broadband spectrally resolved absorption detector (Fig. 2b). While the spectral output of the PX light source extends into the near infra-red, the instrument response function of the 16-bit spectrometer results in a continuous absorption measurement window of our detector of 180–890 nm. The full emission spectrum of the PX light is shown in Fig. 4a, measured by an on-board miniature spectrometer.

We evaluated the stability of the PX source by monitoring long-term drift in signal intensity across all wavelengths. This was evaluated both on mains power and when powered by a battery source. The PX repetition rate was set to 1 Hz and monitored over time (Fig. 4b, c). No significant drift in total intensity was observed for either mains or battery-powered runs. An average change in intensity of 0.01 ± 0.03% per 10^3^ s (*n* = 3) was measured across all individual wavelengths, the greatest change being measured for redder wavelengths. High energy pulsed light in the deep UV can lead to unintended photodegradation of sample analytes. We examined this by exposing the flow cell filled with a 24-component mixture of PAHs to the full spectrum of the PX light source over a range of intensities for an extended period of time. The pulse rates tested were 1, 2, 100, and 220 Hz equivalent to total energy delivered to the sample of 0.2, 0.4, 20, and 45 µJ s^−1^, respectively. We monitored the full spectral intensity every 10 min while the sample was continuously irradiated for 30 min. The integrated full spectral signal did not vary significantly for any source intensity (Fig. 4d). The residency time of analytes within the flow cell is of course, far less under normal operation, only 1 s for a 150 µL min^−1^ flow rate, for example, and so we do not expect any photodegradation of PAHs to occur in our system.

### Injector evaluation

**Fig. 5:**
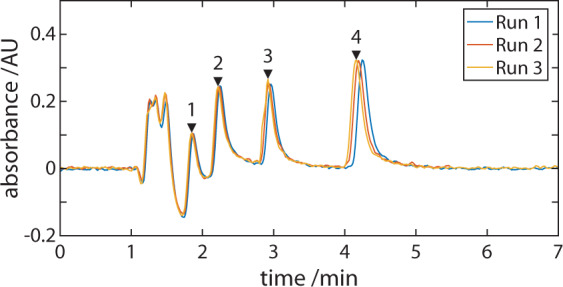
Injector Reproducibility. Chromatographs obtained for the separation of the isocratic certified test mixture 48270-U. Numbers on the chromatogram indicate peak IDs. Table 1 lists percentage RSD values for components of the 48270-U mix separation.

An important aspect in achieving quantitative portable HPLC is precision injection. We proceeded to evaluate the system’s ability to perform reproducible chromatographic runs by first evaluating the reproducibility of the automated constant volume injector assembly. The injector is composed of a geared valve with a wetted area with broad chemical compatibility and a swept volume of 60 nL and switching speed of less than 1 s (see Supplementary Fig. 3). The maximum pressure rating of the valve was 340 bar which is sufficient for use in an HPLC system. We sequentially injected a simple 4-component reference test mixture. Reproducibility was determined by the retention time and peak percentage relative standard deviations (RSDs) ranged from 0.55–0.93% to 1.42–2.20%, respectively (Fig. 5 and Table 1). These compared favourably with separations repeated on a commercial HPLC system (Agilent 1260/90). These results provided confidence that our portable system achieves reproducible sample injection times and injection loadings with performance similar to a conventional, commercial laboratory-based chromatography system.

**Table 1:** Injector Reproducibility.

Peak ID	Time RSD (%)	Area RSD (%)
Injection peak	0.35	1.86
1	0.55	1.42
2	0.78	1.67
3	0.80	2.20
4	0.93	1.91

The isocratic certified test mixture 48270-U was used to measure injector reproducibility. Table lists percentage RSD values for components of the 48270-U mix separation. Peak IDs refer to chromatogram labels in Fig. 5.

### Chromatographic performance, and comparison with a lab-based commercial instrument

**Fig. 6:**
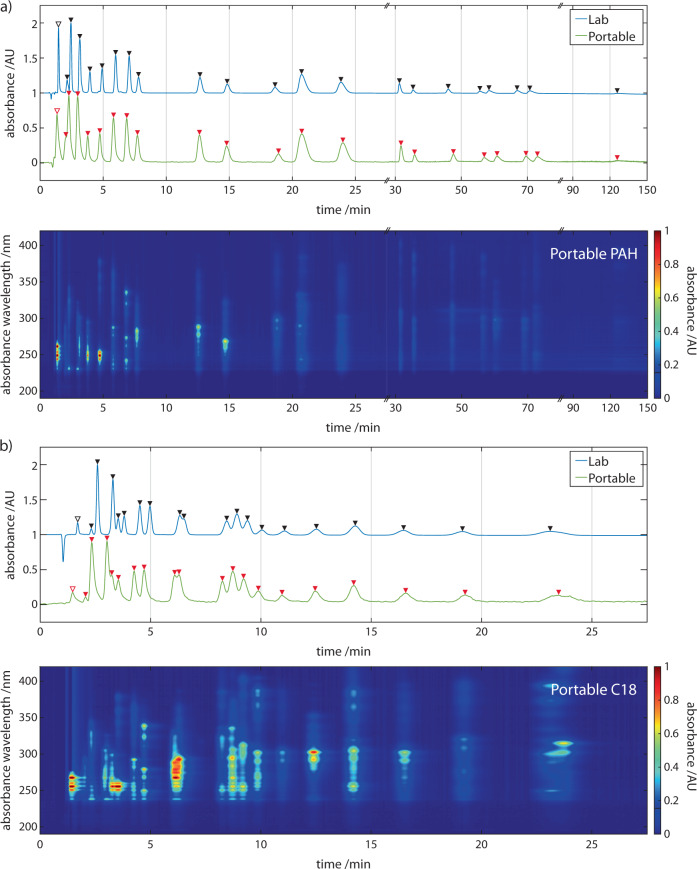
Comparing chromatographic separation of PAHs in commercial & portable instruments. Overlay of PAH mix runs for the portable (green line) and Agilent1260/90 (blue line) systems monitored at 230 ± 2 nm. Arrowheads indicate identified peaks. **a** For Zorbax column runs operating, flow rates were 238 ± 0.35 μL min^−1^ and 236.5 ± 3.3 μL min^−1^ for chromatographic runs on the Agilent1260/90 and portable systems, respectively. **b** For Poroshell column runs, flow rates were 131 ± 0.22 μL min^−1^ and 131 ± 0.18 μL min^−1^ for chromatographic runs on the Agilent1260/90 and portable systems, respectively. **b** Full-spectrum chromatograms ‘heatmaps’ are shown below each 2D trace. Further tabulated results of the PAH mix separation for the portable and Agilent1260/90 systems can be found in the ESI (n = 3) and show the portable system compares favourably. Open and closed arrowheads indicate injection and analyte peaks, respectively.

We proceeded to evaluate the chromatographic performance of the portable system to separate a 24-component certified reference mixture of PAHs. The portable and commercial instruments differ in part due to the dimensions of their connective fluidic tubing and resultant void volumes between components. Nevertheless, in order to quantitatively compare their performance, we used the same microbore columns consecutively in each system. Comparative runs were performed on two microbore columns, the Zorbax Eclipse PAH column (2.1 × 100 mm, 3.5 μm particle size) and the Poroshell 120 EC-C18 (2.1 × 50 mm, 2.7 μm particle size) column. The former column is optimised specifically for the separation of PAHs and therefore is ideal for validation studies of the downstream analysis applied. The latter column provides significantly faster elution of the PAH species, at the cost of species resolution, and is therefore more suited for field acquisitions. The PAH mix was injected (5 µL at 50 ng/µL of each species for ‘Zorbax’ runs and 5 μL at 5 ng/µL of each species for ‘Poroshell’ runs) and exemplar single wavelength chromatograms at 230 ± 2 nm are shown in Fig. 6a. In both chromatograms fewer peaks (19 and 21, respectively) out of an anticipated 24 are resolved.

To permit the identification of analytes in complex mixtures by their characteristic retention times (RT), separations with sufficient reproducibility are necessary. For ‘Poroshell’ runs, the variation of RTs ranged from 0.09 to 0.57% RSD on the portable and 0.07–0.74% RSD on the conventional system. For ‘Zorbax’ runs, the variation of RTs ranged from 2.15 to 4.54% RSD on the portable and 0.98–2.84% RSD on the conventional system. (see Supplementary Tables 2 and 3 for full tables). There is good agreement of the retention times for all species eluted and absolute differences between the systems are attributed to the minor differences in flow rates. From these results, it appears that the separation capability of the two systems is comparable and, encouragingly, suggests that methods developed on the commercial system may be straightforwardly implemented in the portable instrument with little or no development work required.

PAH mixtures are notoriously difficult to completely resolve spatially by HPLC methods alone, even with the use of specialised columns^47^. Given PAHs typically arise as multi-species mixtures, the effects of this issue need to be addressed if a portable system is to be used to characterise, classify and quantify PAH species in the field. Notable reports combining gradient-based separations and superficially porous particulates to enable fast LC have been made^48^. While our separation is comparable to our lab-based experiments, not all features are resolved. Gradient-based methods could enhance separation performance but would increase the complexity of the system and limit its portability. We explored how we could solve these issues using spectral absorption detection with high-pressure liquid chromatography. On our portable system, we were able to obtain ‘full spectrum’ chromatographs of the 24-component PAH mix with spectra spanning 180–890 nm and 0.217 nm optical resolution at a rate of 1 Hz up to a maximum of 220 Hz (Fig. 6). This is the first report of a portable HPLC being able to detect analytes down to 180 nm and being capable of acquiring full-spectrum data. As can be observed from the heatmap of the full spectrum chromatographs in Fig. 6, PAHs are more active in the 230–300 nm spectral region and individual spectra absorption can extend beyond 400 nm. At 230 ± 2 nm we observe separated peaks with good SNR; however, due to their differing absorption spectral profiles, there are less congested regions that would allow the baseline separation of specific analytes due to the disappearance of neighbouring peaks.

### Spectral deconvolution of hidden peaks

Our goal was to spectrally classify all detectable species in the mixture. Therefore, it was necessary to first determine the total number of species detected and, in doing so, deconvolve overlapping and hidden peaks. The concentration profile of an eluting species is expected to be a Gaussian centred at its characteristic retention time. Deconvoluting combinations of these Gaussians (Gaussian Mixture Models, GMMs) can be a straightforward approach to determine the number of distinct species occupying spatially unresolved peaks. However, GMMs can be problematic since they are prone to overfitting and interpretations can lead to more components in a model than is supported by the data. Another problem is exemplified in our PAH data. For instance, for a set of single wavelength chromatograms in this dataset, both the two- and three-species GMMs fit the peaks eluting between 5.0 and 7.0 min (Fig. 6b) with similar accuracy. When fitting a two-species GMM to the data, the estimated retention time of the leading peak varies depending on which wavelength (244–374 nm) the chromatogram is taken. This is not what is expected for a true two-species set. This shift arises due to the varying relative contributions of the species within the overlapping peaks. The GMM accommodates the altering shape of the chromatographic trace at each wavelength by varying the retention time to improve the overall goodness of fit for the peaks.

**Fig. 7:**
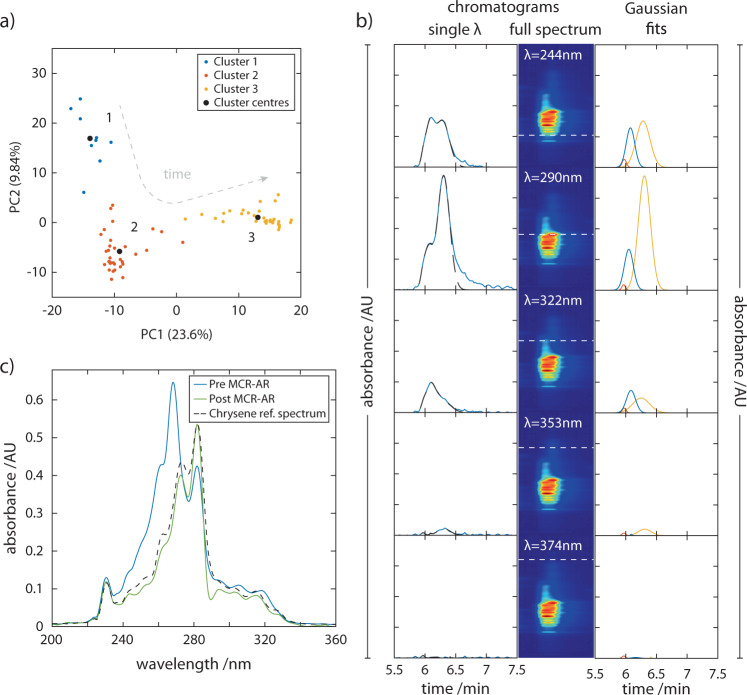
Spectral deconvolution of hidden peaks. **a** The number of species is estimated using a PCA plot and the K-means clusters of the spectra obtained within the elution time range 5.0–7.0 min, suggest the presence of 3 unique species. The black circles denote the centre of each cluster. **b** Estimating the retention times of each analyte using a 3 species Gaussian mixture model (GMM). Left column: Each chromatogram (blue line) plotted corresponds to a detection wavelength window of ±2 nm at the wavelengths indicated in the central column. Dashed black line indicates the sum of the GMM. Central column: The full spectrum chromatogram from Fig. 6b) is cropped to correspond to the time range of interest. The dotted white lines indicate the specific wavelengths denoted in white text. Right column: Results of the GMM for each absorption wavelength. **c** The spectrum of central hidden peak is convolved with that of the flanking peaks (Pre MCR-AR spectrum). The deconvolved spectrum (Post MCR-AR) is the result deconvolution by MCR-AR. Based on the order of elution, a reference spectrum of chrysene is shown for comparison.

A portable HPLC system capable of only single wavelength absorption detection would be restricted here and no further analysis would be possible. However, using multiple wavelength chromatograms, we are able to elucidate the number of species with improved confidence. Performing principal components analysis (PCA) over the full spectrum chromatograph within this time region revealed three dominant K-means clusters, as determined with the elbow method, suggesting 3 unique species (Fig. 7a). Complementary LC-MS data indicates that all three species within this region share the same molecular weight (228 Da) and peaks estimated to correspond to the elution of benzo[c]phenanthrene, chrysene, and benz[a]anthracene found within the mixture (see Supplementary Table 2). To minimise the contribution of the hidden peak, we inspected spectra obtained at the early-onset (rising) and late-end (trailing) timepoints of the chromatographic trace for these peaks; we defined this as the time point at which the absorbance was 10% that of the rising or trailing peak apparent maximum absorbance. These spectra showed good correspondence to reference spectra of benzo[c]phenanthrene (*r*^2^ = 0.90 ± 0.02), rising peak, and benz[a]anthracene (*r*^2^ = 0.92 ± 0.01, ref spectrum^49^:), trailing peak (see below for details on the classification method). The hidden peak could therefore be assumed to be chrysene but confirmation by spectral classification would be preferred. However, for the case of the hidden peak’s elution in this separation there was no timepoint where its UV–vis spectrum was not convolved with the two flanking species. The spectrum obtained at the GMM model derived R_t_ of Chrysene produced a convolved spectrum with low similarity to reference spectra (*r*^2^ = 0.79 ± 0.01, ref spectrum^49^:). Thus, spectral deconvolution was necessary to resolve and classify this analyte.

To achieve this, Multivariate Curve Resolution with Alternating Regression (MCR-AR) was implemented to obtain deconvoluted spectra^50^. To generate accurate initial estimates of the spectra for each species, the peaks were first fitted with a three-species GMM across all wavelengths, informed by the results of the PCA analysis (Fig. 7b). Choosing spectral regions where a subset of only 1 or 2 peaks were present allowed the determination of the retention times (R_t_) for each of the 3 species. Fixing all R_t_, the three-species GMM was used to determine the relative contribution of each species to the signal as a function of time. This guided the initial estimates of the rising and trailing peak spectra to be at timepoints which maximised any single species SNR, while also minimising the contribution of interfering neighbouring species. For the initial guess of the hidden species, the initial estimate of its spectrum was set to the spectra collected at its R_t_, as determined by the GMM.

MCR is a chemometric method for analysing mixtures in order to determine the relative abundances and species signatures within a chemical mixture. Formally known as ‘endmember extraction’, MCR may be utilised here for spectral unmixing or spectral deconvolution enabling the extraction of spectra and relative concentration of each of the contributing analytes, particularly to determine the spectrum of the hidden peak (Fig. 7c). We used MCR with alternating regression (MCR-AR) to obtain deconvoluted spectra and was found to significantly increase the similarity score of the hidden peak spectrum to that of chrysene; prior to spectral deconvolution, the spectrum obtained at the chrysene R_t_ timepoint, when compared to a reference spectrum it exhibited an *r*^2^ value of 0.79 ± 0.01 and a Fréchet distance of 0.30 ± 0.02 (*n*_chroms_ = 3). After spectral deconvolution, the spectrum obtained had an improved *r*^2^ value of 0.96 ± 0.03 and a Fréchet distance of 0.27 ± 0.01 (*n*_chroms_ = 3), when compared to the same standard. This approach was found to be consistent in recovering the spectrum reproducibly—the deconvoluted spectra of chrysene across each run, when compared amongst themselves, exhibited a *r*^2^ value of 0.97 ± 0.02 and a Fréchet distance of 0.036 ± 0.03, suggesting that all deconvoluted spectra across all chromatographic runs had a high degree of similarity.

Additional instances where co-eluting species are identified and their spectra deconvolved can be found in the Supplementary Information (see Supplementary Figs. 4 and 5).

### Classification of species using spectral fingerprinting

In the field, unsupervised real-time results offer the ability for users to make decisions instantly. Compared to using RTs exclusively to identify species, using spectral fingerprinting is a more robust and discriminating approach, suited to complex or ‘messy’ samples, where matrix effects can unexpectedly interfere with the elution of species of interest. We investigated the ability of the full spectrum system in conjunction with machine learning techniques to enable the real-time classification of PAHs. In order to classify species, it was necessary to curate a database of reference spectra. To achieve this, we data-mined spectra firstly from exclusively online sources, including literature published spectra and public databases. In addition, using automatic extraction techniques we were also able to process graphical figures of spectra in the literature to obtain the underlying numerical data^51,52^, The motivation to this approach was to explore how the system could cope in classifying species without the pre-curation of a bespoke or specialised spectral database. This would allow the platform to be more adaptable to different applications and help minimise the time and cost for rapid deployment. The source of all spectra, and extraction/processing procedures used, here can be found in Supplementary Table 4.

**Fig. 8:**
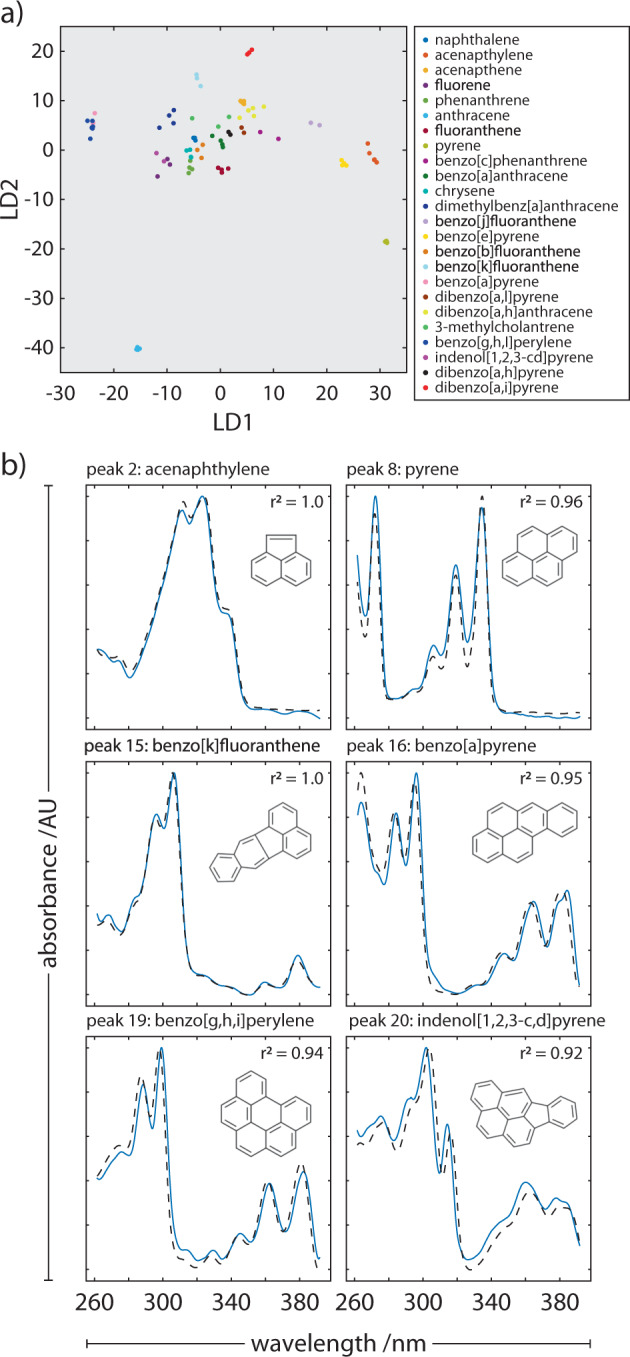
Spectral fingerprinting of analytes. **a** Linear discriminant analysis of the spectra found in each chromatographic peak and their assignments, including deconvoluted hidden peaks. **b** Peak spectra (blue continuous line) and reference spectra of the matched species (black dashed line) of classified species using the species LDA classifier described above. The similarity (*r*^2^ value) between the spectra observed for the peaks identified, and reference spectra is noted in each plot. Matched spectra of all species may be found in the Supplementary Information (see Supplementary Figs. 6 and 7).

The reference spectra (*n* = 64, literature only) were obtained from various sources with differing instrument configurations/conditions, such as spectral window and resolution, and covered 21 of the total 24 PAH species in the test mixture. In order to use them to classify species using spectra obtained on the portable instrument, we processed the data as follows: the reference spectra were linearly interpolated to the same wavelength pixel spacing as the on-board spectrometer (Δλ: 0.217 nm) and smoothed using a Savitzky–Golay filter (Polynomial order: 5, window length: ±4.5 nm) to reduce noise artefacts arising from the digitisation process. The spectra were then differentiated to their first derivative in order to remove any baseline offset. The processed spectra spanned a total wavelength range of 177–730 nm, where the majority of spectra spanned the range approximately 200–450 nm. A linear discriminant analysis (LDA) model was then trained using the reference spectra to find a linear combination of spectral features that would characterise the different species (Fig. 8). This was then used to classify spectra obtained on the portable instrument. For non-congested chromatographic peaks, the spectrum used for each species was sampled at the timepoint of maximum absorbance. For overlapping and hidden peaks, deconvolved spectra were obtained using methods described earlier. The absorbance spectra were pre-processed by smoothing (see above) before undergoing classification using the LDA model. Due to the small sample size of reference spectra available for training, a ‘tiebreak’ stage was introduced to the classifier to account for any species whose LDA classification probability was inconclusive; candidate species undergo an *r*^2^ similarity comparison in order for a final decision to be made. Matched spectra of all species may be found in the Supplementary Information (see Supplementary Figs. 6 and 7).

**Fig. 9:**
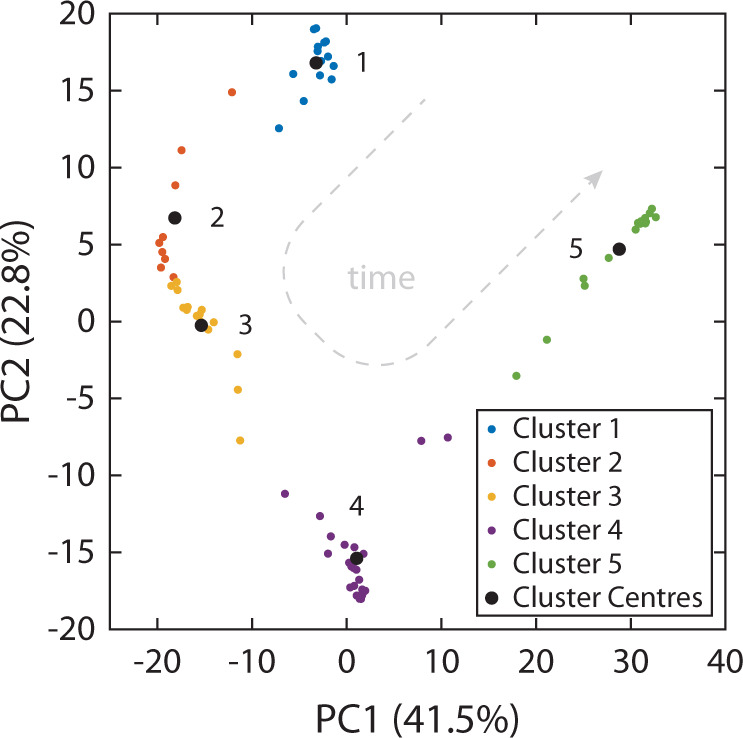
Spectral deconvolution of hidden peaks. The number of species is estimated using a PCA plot and the K-means clusters of the spectra obtained within the elution time range 8.0–10.2 min, suggesting the presence of 5 unique species. The black circles denote the centre of each cluster. The trajectory of spectra in the space determined by the principal components.

Our process was able to achieve a 100% classification rate for 18 of the 21 species with literature reference spectra. One possibility for the drop in classification score for the remaining PAHs was the few spectra (*n* = 1–2) available in the literature for use in our training dataset. To supplement the training dataset and increase its coverage to species without online spectra, we acquired additional reference spectra using commercially sourced reference standards of PAHs. The PAHs for which spectra were acquired using reference standards are indicated in ESI and are publicly available at the following GitHub repository (https://github.com/AnalyticalSystemsResearch/). The LDA model was re-trained to incorporate the additional spectra (*n* = 88, literature plus newly acquired). When reviewing the new classification scores of Poroshell runs, it was evident that the classification of the peak eluting at 8.72 ± 0.09 min (Fig. 6b) would consistently switch between benzo[b]fluoranthene and benzo[e]pyrene from run to run. On closer inspection of the full spectrum chromatograms, there was evidence of co-elution. K-means clustering of the principal components of the spectra acquired during the elution of the 4 peaks between 8.0 min and 10.2 min suggested 5 dominant clusters when employing the elbow method (Fig. 9). After spectral deconvolution and classification, it was confirmed that benzo[b]fluoranthene and benzo[e]pyrene were, indeed, present, with very close retention times of 8.69 ± 0.07 min and 8.74 ± 0.08 min, respectively. Prior to deconvolution, the convolved spectrum at those retention times exhibited a *r*^2^ similarity to benzo[b]fluoranthene and benzo[e]pyrene of 0.82 ± 0.06 and 0.83 ± 0.05 and Fréchet distances of 0.24 ± 0.01 and 0.37 ± 0.04, respectively. This and the near to complete overlap of their elution peaks explains the initial classification results. After deconvolution, the r^2^ similarities to their corresponding reference spectra increased to 0.89 ± 0.03 and 0.89 ± 0.02 and Fréchet distances were reduced to 0.22 ± 0.01 and 0.31 ± 0.05, respectively. This set of co-eluting peaks was particularly challenging. Despite this, it was encouraging that the process of spectral deconvolution and classification described here was successful across all chromatographic runs.

**Fig. 10:**
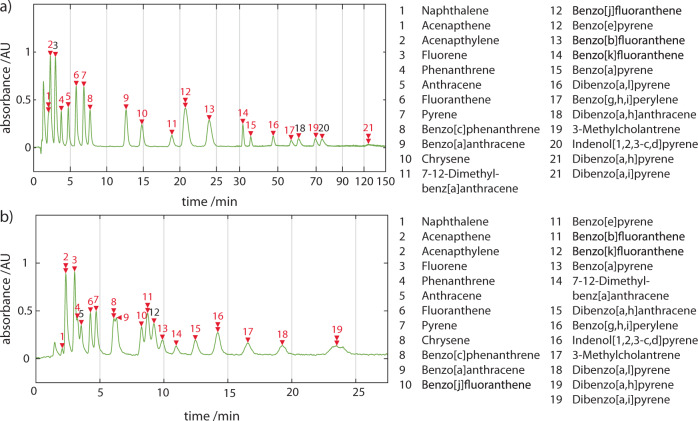
Chromatograms annotated with spectrally identified species. Exemplar chromatograms of the 24 component PAH separation run on the portable LC system at 230 ± 2 nm annotated with peak identification numbers using **a** Zorbax PAH and **b** Poroshell C18 columns. Double arrowheads indicate co-eluting PAHs. The table lists which PAHs elute at the indicated peak number, determined by spectral identification.

Our expanded classifier, that incorporated spectra both sourced from the literature and measured in our laboratory, was able to achieve a 100% classification rate for all 16 PAHs on the United States (US) Environmental Protection Agency (EPA) priority pollutant list and 16 PAHs on the EU ‘15 + 1’ PAHs list. For both Zorbax Eclipse and Poroshell column runs, we achieved an improved classification rate of 100% for 24 of 24 species with reference spectra at these conditions. The fully annotated chromatograms at 230 ± 2 nm are shown in Fig. 10.

The limits of detection varied for each species, with the lowest achievable detection being 2.5 ng/mL to 55.8 ng/mL (see Supplementary Table 5 for details). The method detection limits published by the US EPA for HPLC range from 0.018 ng/mL to 2.3 ng/mL^53^. There are several possibilities when attempting to improve LODs in LC. Here, we expect lower LODs could be achieved by improving sensitivity in the flow cell. The absorption length of the z-cell may be extended further, but at the sacrifice of temporal resolution. The use of optofluidic waveguides would be able to maintain small cell volumes while increasing effective path length and improving light transmission. Additionally, the spectrometer array is optimised for dynamic range not sensitivity. Alternative pixel-arrays chips may be incorporated for applications where sensitivity is a priority. While the PX light source used here is advantageous in terms of its broadband spectrum and high intensity in the deep UV, the development of light sources that maintain this while exhibiting lower noise would be expected to improve LODs further. Pre-treatment methods to isolate and concentrate PAHs will lower effective LODs. While these have focussed on lab-based approaches out of necessity, reports of field-amenable methods have been made. Of note, is the work by Irlam et al. developing a 3D-printed solid-phase extraction system for at-site trace analysis of contaminants and the general outputs of the frugal science field toward the cost-effective translation of lab-based methods^54–56^.

### Testing samples in the field

**Fig. 11:**
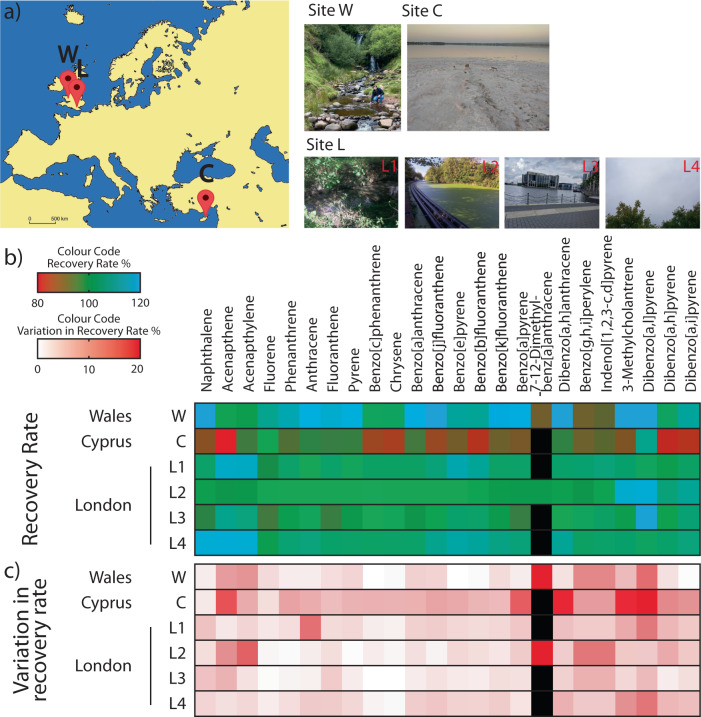
Field testing of water samples using portable LC. **a** A schematic map of Europe indicating the locations of water sources that were tested. W: Wales (1 site), C: Cyprus (1 site), L: London, England (4 sites). Photographs of the site of sample testing are shown. See text for coordinates and details. Colour maps visualising **b** the recovery rates of PAHs and **c**) the variation in the recovery rate of PAHs. Colour codes indicated in figure; black indicates no recovery or value.

To determine the degree to which matrix effects affect the spiked PAHs, we tested the capabilities of the instrument and methods developed above by on-site sampling of various water sources in the United Kingdom (Wales and England) and Cyprus (Fig. 11a). In Wales (Site W), water was sampled from Nant Bwrefwr, a principal tributary of the Caerfanell river at Craig y Fan Ddu in the Brecon Beacons national park (51°50′56.2″N 3°22′19.7″W). In England (Site L), water was sampled at 4 locations in London: L1) the River Pinn in North-West Greater London, L2) the Paddington arm of the Grand Union Canal at Park Royal (51°31′45.0″N 0°15′00.8″W), L3) the Millwall Dock on the River Thames south of Canary Wharf at the Isle of Dogs (51°29′36.6″N 0°00′55.8″W), and L4) rainwater collected in North-West Greater London. In Cyprus (Site C), water was sampled from Larnaca Salt Lake at the north shore near the Salt Lake viewpoint (34°54′20.8″N 33°37′12.0″E). Water samples were spiked with the 24-component PAH mix (5 ng/µL) and injected into the portable LC via a 0.22 μm filter to remove particulates. This would include, for example, sediments of varying granularity in river samples and plant matter from the canal sample. No further processing of samples was carried out in the field. Chromatographic runs were performed as before and the recovery rates of each PAH is calculated and presented in Fig. 11b (see Supplementary Table 6 for numeric values). PAH spike recoveries were found to range from 82.16% to 170.36%, compared to the recovery of PAHs spiked into HPLC grade water. The PAH mix is a certified reference material so errors in the preparation of standard solutions are not expected to contribute significantly to the recoveries deviating from 100%. The measured variations in recovery rates (*n* = 3) did not exceed 5% for the majority of measurements (Fig. 11c, see Supplementary Table 7 for numeric values). Matrix effects can unpredictably alter characteristic retention times and can enhance or depress analyte signal, affecting the accuracy of quantification. Photographs of samples from each of the sites (see Supplementary Fig. 8a) show their turbidity and colouration due to salts, sediment, organic matter, and aquatic life, such as plants and small creatures. We tested these samples using water quality test strips (Supplementary Fig. 8b); the quality of the samples varied mostly in total alkalinity, pH, hardness, and copper content (see Supplementary Table 8 for numeric values). Water salinity is known to affect PAH solubility and may help explain the drop in recovery for the Larnaca Salt Lake sample, though the concentration of the spiked PAHs is well below their solubility limits^57^. 7–12-dimethylbenz[a]anthracene (DMBA) was detected in samples from sites W (river) and L2 (canal), albeit with high variation in its recovery, but not at all from sites C (salt lake), L1 (river), or L3 (river). Due to their relatively low solubilities in water and their lipophilicity, PAHs have a tendency to adsorb onto particulates and sediments. However, it’s not clear why DMBA is singly affected and not the other PAHs. Surprisingly, the recovery rate of the most turbid samples (L1, L2) did not appear to be affected. For all field samples tested, the spectral classification rate was 100% for all detected PAHs, unhindered by any matrix effects of the samples.

## Conclusions

We have reported the first portable HPLC system with a broadband spectral detector. It is a fully stand-alone system with all necessary instrumentation on-board and no additional requirements for its operation. We were able to acquire the ‘full’ UV–vis spectrum of eluting species and have shown how this enables the deconvolution of overlapping peaks to, not only, identify but also classify hidden species. The capability to spectrally resolve overlapping or difficult to chromatographically-resolve peaks greatly extends the scope and applicability of portable chromatography systems. We expect this to prove a powerful tool in the interpretation of chromatographic data obtained in the field. While this report focuses on these techniques as applied to PAH analysis, they are generalisable to other analytes and applications.

Portable chromatography systems have so far been reported in isolation. As far as we are aware, this is the first report comparing the performance of a portable system directly to a conventional laboratory-based counterpart. The portable system compares favourably to a commercial system in tests measuring the flow-rate stability and responsiveness of their high-pressure pumps. Their chromatographic performance also compared favourably when using the same separation method for a 24-component mixture of PAHs (showing the potential to use common methods in lab-based and portable instruments).

We were able to train a species classifier which was able to achieve a 100% classification rate for all PAHs under test here with reference spectra, with complete coverage of PAHs listed in the US EPA’s priority pollutant list. Importantly, a high classification rate could be achieved using spectral fingerprinting alone without considering the characteristic retention time of the PAHs. It is, of course, possible to incorporate characteristic retention times and elution order into the classification model. However, these can be unpredictably altered due to matrix effects. We anticipate that this approach will be important to help overcome such matrix effects that might otherwise preclude reliable measurements of these substances in challenging field samples. In doing so, it stands to reason that our methods would be more robust to interference in the field.

We chose to explore how well PAHs could be classified using a fingerprinting database populated solely with reference spectra digitally extracted from the scientific literature. We believe this capability opens the door to more sophisticated field-based measurements that are not restricted to static chemoinformatic libraries but may dynamically populate databases using the chemistry literature while deployed in the field. Using the automated extraction techniques employed in this work, vast databases may be created or expanded with minimal time and expense. Of course, data sharing will be important to achieve this and to support these efforts, we have made all our spectra publicly available.

Using the statistical approaches described here, we have been able to implement unsupervised methods to automatically detect and isolate peaks, including those that co-elute or are hidden, and proceed to positively identify and classify species in real-time on board the instrument. We believe the automated and unsupervised detection and characterisation of peaks is a very crucial step in field based analytical methods. We conclude that portable HPLC with broadband absorption detection is a suitable method for the surveillance of PAHs and expect this will enable rapid, on-site decisions to be made independent of a central laboratory.

## Methods

### Reagents

Analytical or HPLC grade solvents and chemicals were used as part of this work. Ultra-pure water was generated in the lab (15 & 18.2 MΩ). The mobile phase was 70:30 (v/v) acetonitrile: water. Reference test mixtures were used in the study; isocratic separation test mixture (48270-U; Merck) and Quebec Ministry of Environment 24 component PAH Mix (H-QME-01; AccuStandard, USA) containing: acenaphthene (83-32-9), acenaphthylene (208-96-8), anthracene (120-12-7), benz(a)anthracene (56-55-3), benzo(b)fluoranthene (205-99-2), benzo(j)fluoranthene (205-82-3), benzo(k)fluoranthene (207-08-9), benzo(g,h,i)perylene (191-24-2), benzo(c)phenanthrene(195-19-7), benz[a]pyrene (50-32-8), benz[e]pyrene (192-97-2), chrysene (218-01-9), dibenz(a,h)anthracene (53-70-3), dibenz[a,h]pyrene (189-64-0), dibenz[a,i]pyrene (189-55-9), dibenz[a,l]pyrene (191-30-0), 7,12-dimethylbenz(a)anthracene (57-97-6), fluoranthene (206-44-0), fluorene (86-73-7), indeno(1,2,3-cd)pyrene (193-39-5), 3-methylcholanthrene (56-49-5), naphthalene (91-20-3), phenanthrene (85-01-8) and pyrene (129-00-0). CAS numbers in brackets. Pure reference standards were also used in this work where required: acenapthylene, acenapthene, 7–12-dimethylbenz[a]anthracene, benzo[c]phenanthrene, benzo[j]fluoranthene, benzo[e]pyrene, dibenzo[a,l]pyrene, dibenzo[a,h]anthracene, dibenzo[a,i]pyrene, 3-methylcholantrene, benzo[ghl]perylene.

### Instrumentation

A commercial HPLC was used in this work (Agilent 1260/90 Infinity II; Agilent, UK) comprising a quaternary pump and a multiwavelength detector with deuterium lamp. The hand-portable anywhereHPLC system was evaluated using the BETTER (portaBle fiEld Testing sTandard framEwoRk) criteria (see Supplementary Fig. 9) and built as follows:

#### Pump

The gas pump assembly comprised a 0.25 L high-pressure (4500 psi/310 bar) tank connected via 316/316 L stainless steel tubing and valves to a 150 mL mobile phase reservoir (Swagelok, UK). Ports allowed the gas and solvent reservoirs to be recharged. The pressurised gas source was a nitrogen (oxygen-free) cylinder (BOC, UK). One advantage of using a regulated pressure source is that the flow rate profile is free of variations associated with the movement of mechanical components.

#### Injector

The injector assembly was made using a six-port, two-position automated valve (Labsmith, USA) and a 5 μL PEEK sample loop. Standard tubing and microtight fittings were used throughout. The valves were connected to a manifold (Labsmith, USA) and via USB to a single board computer. The valve position is electronically controlled by software using manufacturer supplied drivers (Labsmith, USA); switching valve positions allows the sample loop to be loaded with sample (position 1) and subsequently injected onto the column (position 2).

#### Detector

Analytes were detected using UV–vis absorption. A pulsed Xenon light source (Ocean Optics, UK), capable of 1–220 Hz pulse rate and 45 µJ/pulse, was fibre-coupled to a z-type flow cell with a 2 µL internal volume and 10 mm path length (Ocean Optics, UK). A miniaturised spectrometer (Ocean Optics, UK) was coupled directed to the flow cell using a custom-machined retaining assembly. A grating dispersed light across a 3648-pixel array allowing for spectral detection down to 0.2 nm resolution. The detector’s full spectral range was 180–890 nm, limited by the spectrometer.

#### Instrument housing

The system has a form factor of 255 mm (width) × 250 mm (depth) × 126 mm (height). Components were mounted on acrylic sheets, machined using a desktop CNC miller, and secured to the case. Cases were custom-designed and 3D printed using a commercial 3D printer (Ultimaker S5; Ultimaker, UK).

#### Sensors & electronics

On board sensors measured system pressure, mobile phase flow rate, instrument temperature. All systems were electronically controlled using a single board computer (SBC). The display and SBC are attached by manufacturer designed mounting holes using metal spacers and mounted on the custom instrument case. The battery is mounted in the void space on the top layer (Fig. 2b (i)) and connected to the SBC.

#### Chromatography

PAH separations were performed using a 50 mm × 2.1 mm, 2.7 µm (length × inner diameter, packing) C18 microbore column (Poroshell 120 EC-C18; Agilent, UK) and 100 mm × 2.1 mm, 3.5 µm PAH-specialised microbore column (Zorbax Eclipse PAH; Agilent, UK). The mobile phase was 70:30 (v/v) acetonitrile and water, as this is the typical solvent mix for many standardised PAH methods. The same microbore columns were used for experiments on either the portable or conventional HPLC instruments.

#### Data acquisition and processing

Data were recorded using custom code, which was written for Python. Raw data were either analysed using the on-board single-board computer or exported and analysed using custom-written scripts for Python or MATLAB.

## Supplementary information

Supplementary Information

## Data Availability

The data that support this study are available from the corresponding author upon reasonable request. Spectral data are publicly available in GitHub at https://github.com/AnalyticalSystemsResearch/.

## Code availability

All code relating to the acquisition and analysis of data is available from the corresponding author upon reasonable request.
