# Supplementary material for: Hand-portable HPLC with broadband spectral detection enables analysis of complex polycyclic aromatic hydrocarbon mixtures
Source: Commun Chem. 2021 Feb 16;4:17. doi: 10.1038/s42004-021-00457-7 (PMC9814556; doi:10.1038/s42004-021-00457-7)
Supplement: Supplementary file 1 — Supplementary Information [file 42004_2021_457_MOESM1_ESM.pdf]

Hand-portable HPLC with broadband spectral detection enables analysis of complex polycyclic aromatic hydrocarbon mixtures

SUPPLEMENTARY INFORMATION

Stelios Chatzimichail[1], Faraz Rahimi[1,2], Aliyah Saifuddin[1,2],

Andrew J. Surman[2], Simon D. Taylor-Robinson[1] and Ali Salehi-Reyhani[1,3*]

[1] Dept. of Surgery and Cancer, Imperial College London, London, W12 0HS, UK

[2] Department of Chemistry, King's College London, London, SE1 1DB, UK

[3] Institute of Molecular Sciences & Engineering, Imperial College London, London, SW7 2AZ, UK

Table of Contents

Supplementary Table 1 Polycyclic aromatic hydrocarbons tested in this work 2

Supplementary Figure 1 Waste Reservoir 5

Supplementary Figure 2 Long term flow rate stability 7

Supplementary Figure 3 Flow rate profile during automated sample injections 8

Supplementary Table 2 PAH peak retention times (Poroshell column runs) 9

Supplementary Table 3 PAH peak retention times (Zorbax column runs) 10

Supplementary Figure 4 Spectral Deconvolutions (Poroshell column runs) 11

Supplementary Figure 5 Spectral Deconvolutions (Zorbax column runs) 13

Supplementary Table 4 Literature reference spectra for spectral fingerprinting 14

Supplementary Figure 6 Classifier matched spectra (Poroshell column runs) 15

Supplementary Figure 7 Classifier matched spectra (Zorbax column runs) 16

Supplementary Table 5 Limits of detection for standalone PAH species 17

Supplementary Table 6 Field sample results – spike PAH recovery rate 18

Supplementary Table 7 Field sample results – variation in spike PAH recovery rate 19

Supplementary Figure 8 Field sample results – sample quality 20

Supplementary Table 8 Field sample results – sample quality 21

Supplementary Figure 9 Evaluation of portable instrument using the BETTER criteria. 22

Supplementary References 23

Supplementary Table 1 Polycyclic aromatic hydrocarbons tested in this work

Name	CAS no.	Molecular Weight (Da)	Molecular Structure
Naphthalene	91-20-3	128	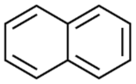
Acenaphthylene	208-86-8	152	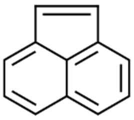
Acenaphthene	83-32-9	154	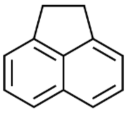
Fluorene	86-73-7	166	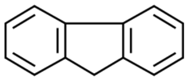
Phenanthrene	85-01-8	178	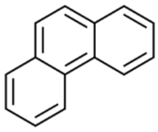
Anthracene	120-12-7	178	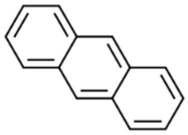
Fluoranthene	206-44-0	202	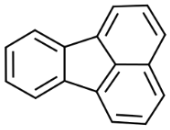
Pyrene	129-00-0	202	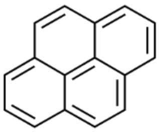
Benzo[c]phenanthrene	195-19-7	228	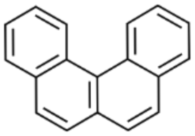

Chrysene	218-01-9	228	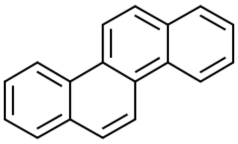
Benzo[a]anthracene	56-55-3	228	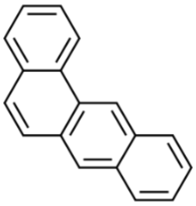
Benzo[j]fluoranthene	205-82-3	252	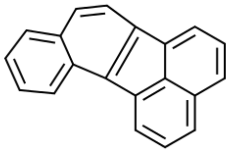
Benzo[e]pyrene	192-97-2	252	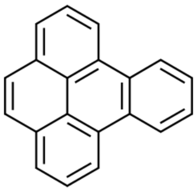
Benzo[b]fluoranthene	205-99-2	252	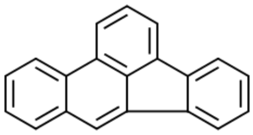
Benzo[k]fluoranthene	207-08-9	252	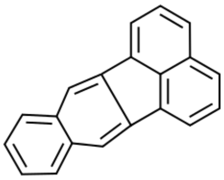
Benzo[a]pyrene	50-32-8	252	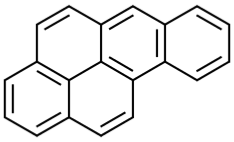

7-12-<br>Dimethylbenz[a]anthracene	57-97-6	256	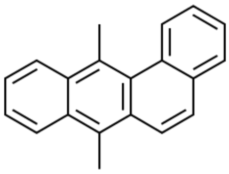
Dibenz[a,h]anthracene	53-70-3	278	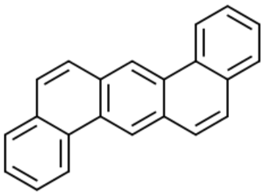
Benzo[g,h,i]perylene	191-24-2	276	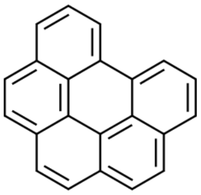
Indenol[1,2,3-cd]pyrene	193-39-5	276	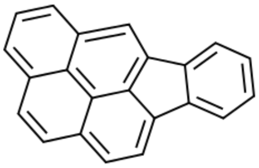
3-Methylcholantrene	56-49-5	268	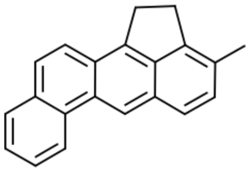
Dibenzo[a,l]pyrene	191-30-0	302	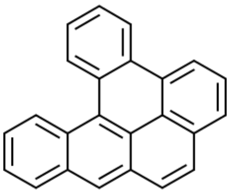
Dibenzo[a,h]pyrene	189-64-0	302	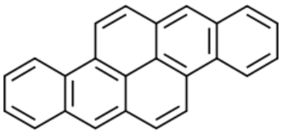
Dibenzo[a,i]pyrene	189-55-9	302	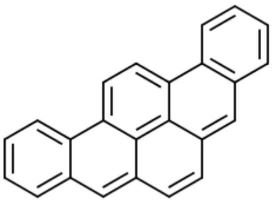

Supplementary Figure 1 Waste Reservoir

The waste reservoir stores solvent waste, which cannot be safely disposed of in the field. The container is 3D printed (Ultimaker S5; Ultimaker, UK) using chemically compatible thermoplastics. When using acetonitrile, co-polyester (CPE) filament (Ultimaker, UK) is compatible, but care must be taken for other solvents which may be incompatible, leading either to swelling or complete breakdown of the container walls. This is demonstrated by immersing 3 commonly available thermoplastic filaments in 100% acetonitrile for 24 hours without agitation (**Supplementary Figure 1a**). Polylactic acid (PLA) is completely degraded, co-polyester (CPE) filament swells, and nylon does not show signs of swelling. The swelling factor is the ratio of the weight of each filament piece before and after immersion. More extensive reports of solvent compatibility may be found in the literature.[12] Since the reservoir stores waste solvent, leaching of material is not necessarily a critical consideration as if it were used to store mobile phase or sample solutions. Nevertheless, compromising the structural integrity of the reservoir is undesirable. Polyether ether ketone (PEEK) has broad chemical compatibility and is recommended for most LC applications; however, it requires specialist printers. The waste reservoir has 3 ports (**Supplementary Figure 1b**), 1) waste from the column line, 2) waste from the sample injector, and 3) a drain port. The outer dimensions are 74 mm × 54 mm × 47 mm (width × depth × height). The volume of waste solvent in the container is estimated from the amount of solvent used during each run and drained periodically using a syringe connected to a luer lock check valve (IDEX, USA). The waste reservoir can include quick-release connectors on the waste in ports so that it may be decoupled from the device and stored for safe disposal. When operating in the field, we found that draining the on-board waste reservoir to a separate container was more straightforward.

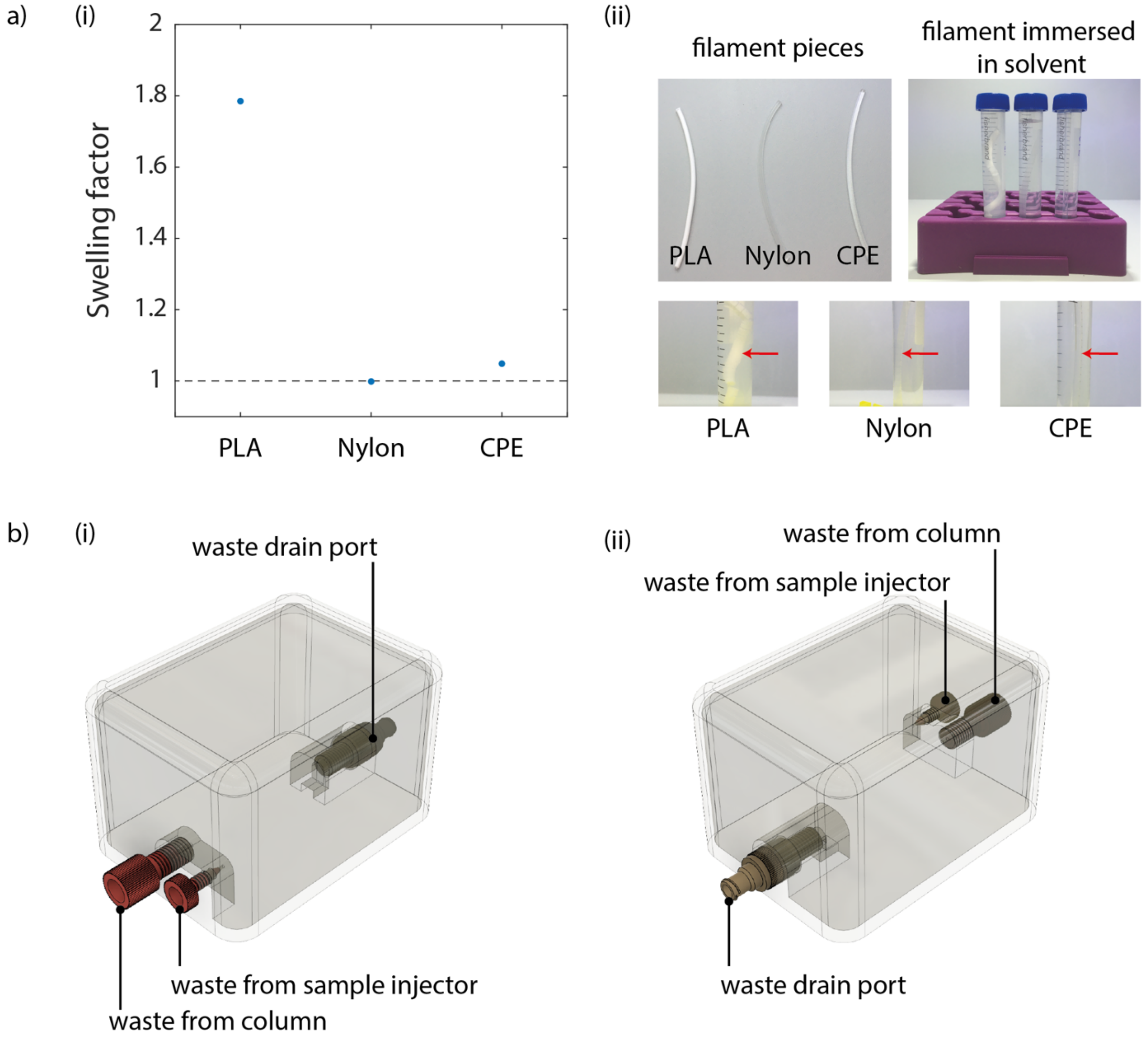
a)
(i)
Swelling factor
2
1.8
1.6
1.4
1.2
1
PLA
Nylon
CPE
(ii)
filament pieces
filament immersed in solvent
PLA
Nylon
CPE
PLA
Nylon
CPE
b)
(i)
waste drain port
waste from sample injector
waste from column
(ii)
waste from column
waste from sample injector
waste drain port

Supplementary Figure 2 Long term flow rate stability

The variation in flow rate was measured over 6 hours, The average flow rate is 149.8 to match a nominal flow rate of 150 μL min^{-1} set on a commercial HPLC driven by a quaternary pump (1260 Infinity II Quaternary Pump; Agilent, UK). An absolute standard deviation about the mean of 0.12 μL min^{-1} was measured. The total time measured is equivalent to 13 sequential 24 component PAH separations using a 2.1 x 50 mm, 2.7 μm particle size Poroshell column (see **Fig. 6** and **Fig. 10** in the main text).

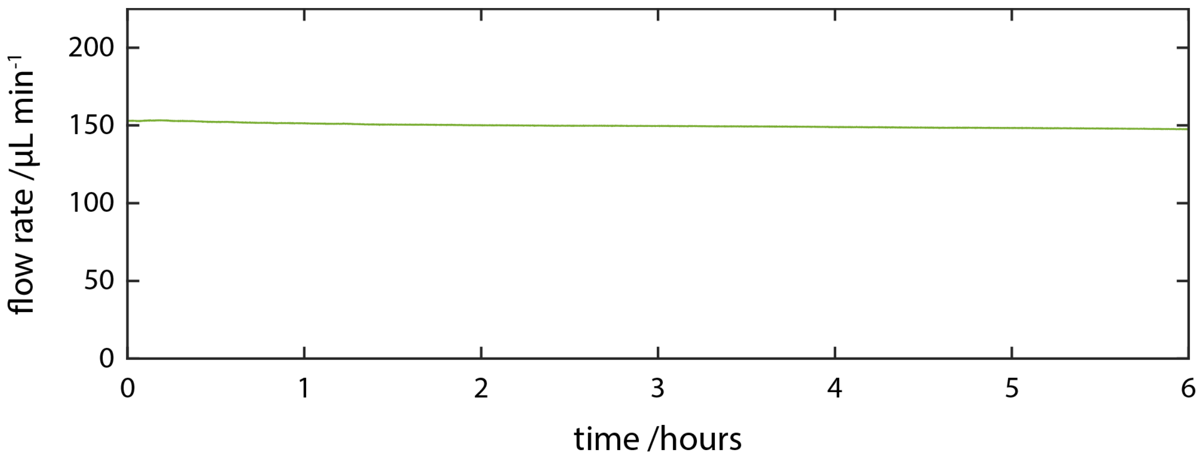

Supplementary Figure 3 Flow rate profile during automated sample injections

Flow Profile of a chromatographic run using the portable system. The inset shows the flow profile during the 'loading' and 'injection' stages of the 6-port injection valve used. In all cases observed, the flow rate value is restored within 1s.

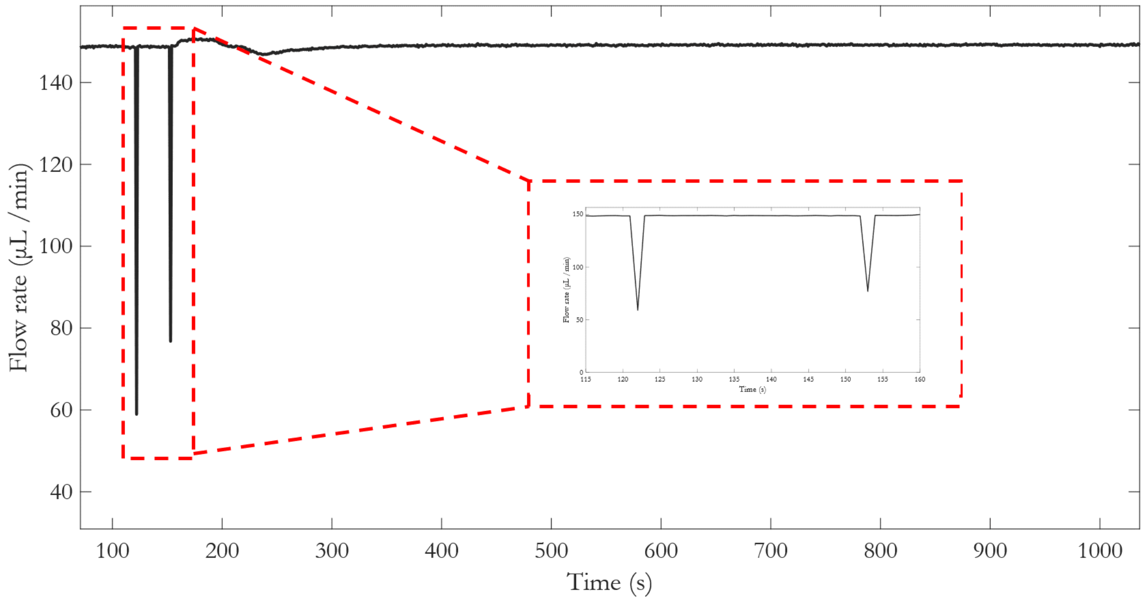

Supplementary Table 2 PAH peak retention times (Poroshell column runs)

Peak retention times (R_t) and peak retention time RSDs for all identified peaks when separating 24 component PAH mixture. Chromatographic separations run using the Poroshell column on the *anywhereHPLC* portable and Agilent 1260/1290 HPLC instruments. For each peak ID, the fingerprinted species and the LC-MS predominant molecular ion are listed.

**Peak ID**	**Matched Species**	**Retention time Portable (min)**	**Retention time Agilent1260 (min)**	**Time %RSD (portable)**	**Time %RSD (Agilent 1260)**	**LC-MS ESI molecular ion (m/z)**
Peak 1	Naphthalene	2.02	2.34	0.40	0.25	128
Peak 2	Acenapthene<br>Acenapthylene	2.32	2.61	0.33	0.21	152<br>154
Peak 3	Fluorene	2.98	3.31	0.57	0.63	166
Peak 4	Phenanthrene	3.23	3.56	0.27	0.74	178
Peak 5	Anthracene	3.51	3.82	0.24	0.50	178
Peak 6	Fluoranthene	4.23	4.54	0.20	0.45	202
Peak 7	Pyrene	4.68	4.99	0.18	0.42	202
Peak 8	Chrysene<br>Benzo[c]phenanthrene	6.08	6.36	0.29	0.33	228
Peak 9	Benzo[a]anthracene	6.27	6.55	0.10	0.40	228
Peak 10	Benzo[j]fluoranthene	8.24	8.49	0.33	0.07	252
Peak 11	Benzo[e]pyrene<br>Benzo[b]fluoranthene	8.72	8.95	0.09	0.17	252
Peak 12	Benzo[k]fluoranthene	9.22	9.42	0.21	0.16	252
Peak 13	Benzo[a]pyrene	9.88	10.10	0.20	0.23	252
Peak 14	7-12-<br>Dimethylbenz[a]anthracene	10.99	11.09	0.18	0.09	256
Peak 15	Dibenzo[a,h]anthracene	12.48	12.56	0.26	0.21	278
Peak 16	Benzo[g,h,i]perylene<br>Indenol[1,2,3-cd]pyrene	14.20	14.32	0.14	0.11	276
Peak 17	3-methylcholantrene	16.57	16.49	0.13	0.19	268
Peak 18	Dibenzo[a,l]pyrene	19.26	19.20	0.12	0.23	302
Peak 19	Dibenzo[a,h]pyrene<br>Dibenzo[a,i]pyrene	23.39	23.25	0.30	0.47	302

Supplementary Table 3 PAH peak retention times (Zorbax column runs)

Peak retention times (R_t) and peak retention time RSDs for all identified peaks when separating 24 component PAH mixture. Chromatographic separations run using the Poroshell column on the *anywhereHPLC* portable and Agilent 1260/1290 HPLC instruments. For each peak ID, the fingerprinted species and the LC-MS predominant molecular ion are listed.

Peak ID	Matched Species	Retention time Portable (min)	Retention time Agilent1260 (min)	Time %RSD (portable)	Time %RSD (Agilent 1260)
Peak 1	Naphthalene<br>Acenapthene	1.97	2.07	4.00	1.22
Peak 2	Acenapthylene	2.17	2.36	2.54	0.98
Peak 3	Fluorene	2.89	3.03	2.15	1.16
Peak 4	Phenanthrene	3.66	3.80	3.41	1.45
Peak 5	Anthracene	4.61	4.72	3.70	1.41
Peak 6	Fluoranthene	5.67	5.73	3.46	1.88
Peak 7	Pyrene	6.70	6.76	3.38	1.72
Peak 8	Benzo[c]phenanthrene	7.54	7.48	3.54	1.67
Peak 9	Benzo[a]anthracene	12.40	12.08	3.44	2.49
Peak 10	Chrysene	14.53	14.10	3.40	2.47
Peak 11	7-12-<br>DimethylBenz[a]anthracene	18.05	17.77	4.31	2.08
Peak 12	Benzo[j]fluoranthene<br>Benzo[e]pyrene	20.57	19.73	3.46	2.63
Peak 13	Benzo[b]fluoranthene	23.79	22.70	3.42	2.67
Peak 14	Benzo[k]fluoranthene	31.40	29.72	3.40	2.84
Peak 15	Benzo[a]pyrene	35.48	33.76	3.53	2.55
Peak 16	Dibenzo[a,l]pyrene	47.07	43.95	3.80	2.08
Peak 17	Benzo[g,h,i]perylene	56.39	53.08	3.79	2.29
Peak 18	Dibenzo[a,h]anthracene	60.16	55.59	4.28	2.70
Peak 19	3-methylcholantrene	68.83	63.72	4.54	2.48
Peak 20	Indenol-cd-pyrene	72.31	67.35	4.37	2.44
Peak 21	Dibenzo[a,h]pyrene<br>Dibenzo[a,i]pyrene	129.33	125.84	12.40	9.43

Supplementary Figure 4 Spectral Deconvolutions (Poroshell column runs)

Peak 2

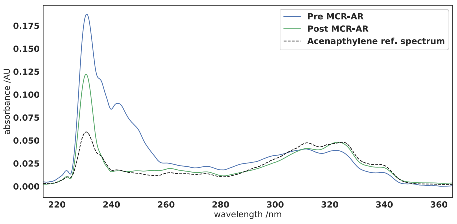

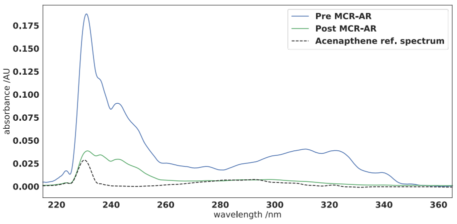

Peak 8

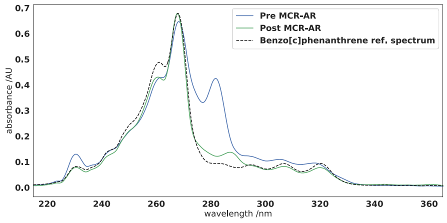

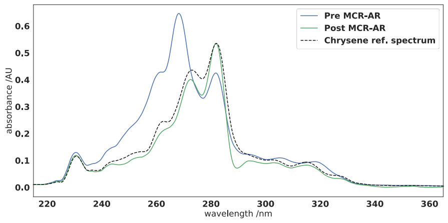

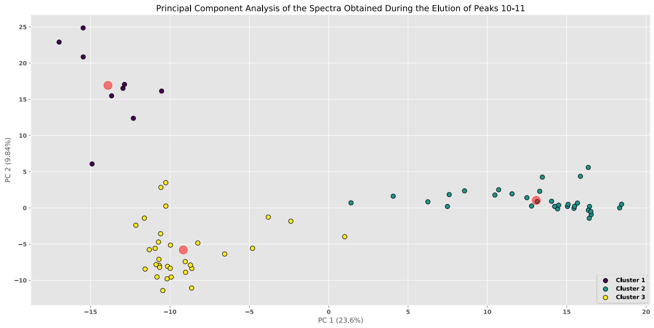

Peak 11

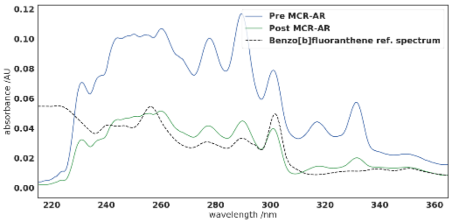

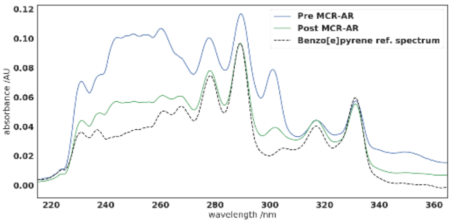

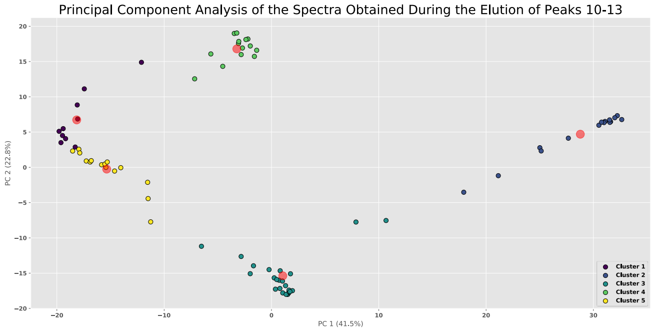

Peak 16

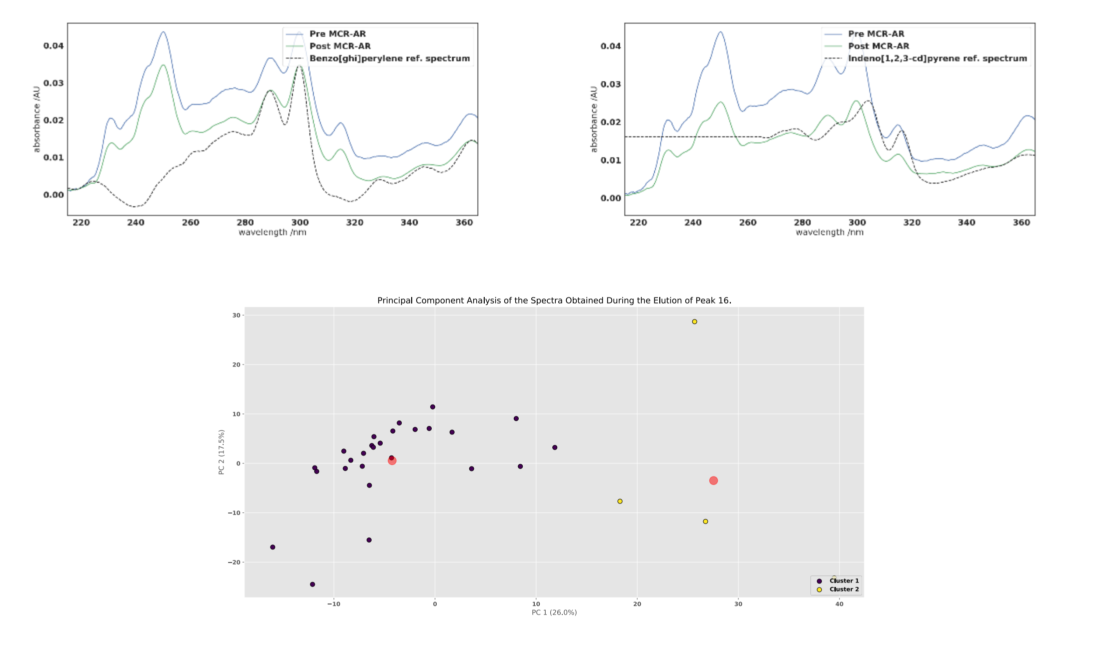

Peak 19

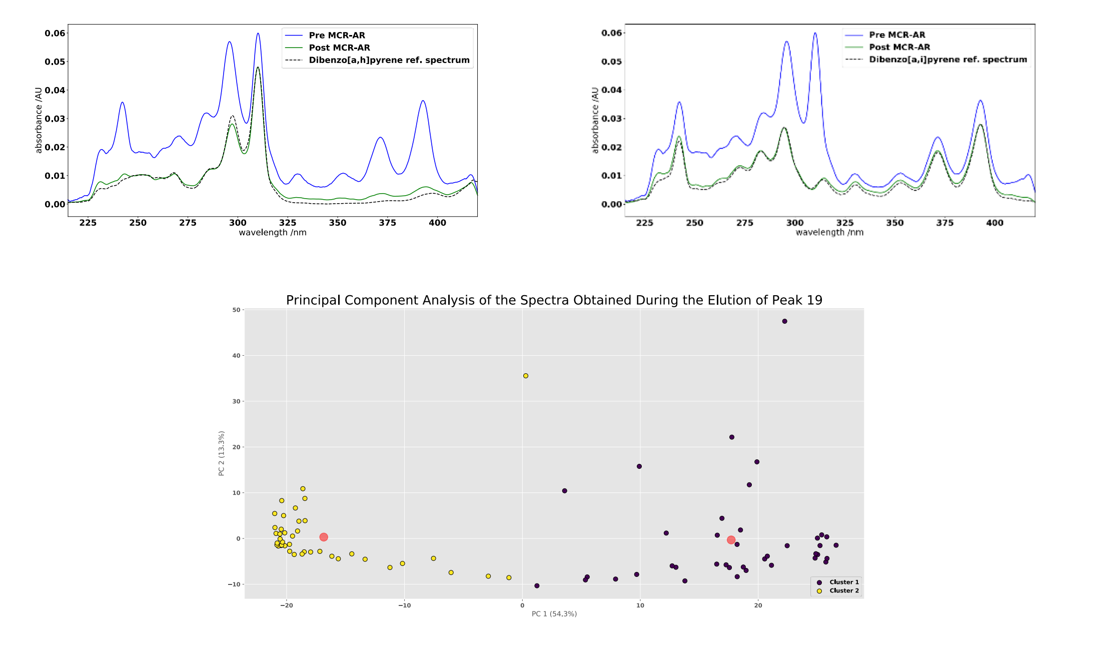

Supplementary Figure 5 Spectral Deconvolutions (Zorbax column runs)

Peak 13

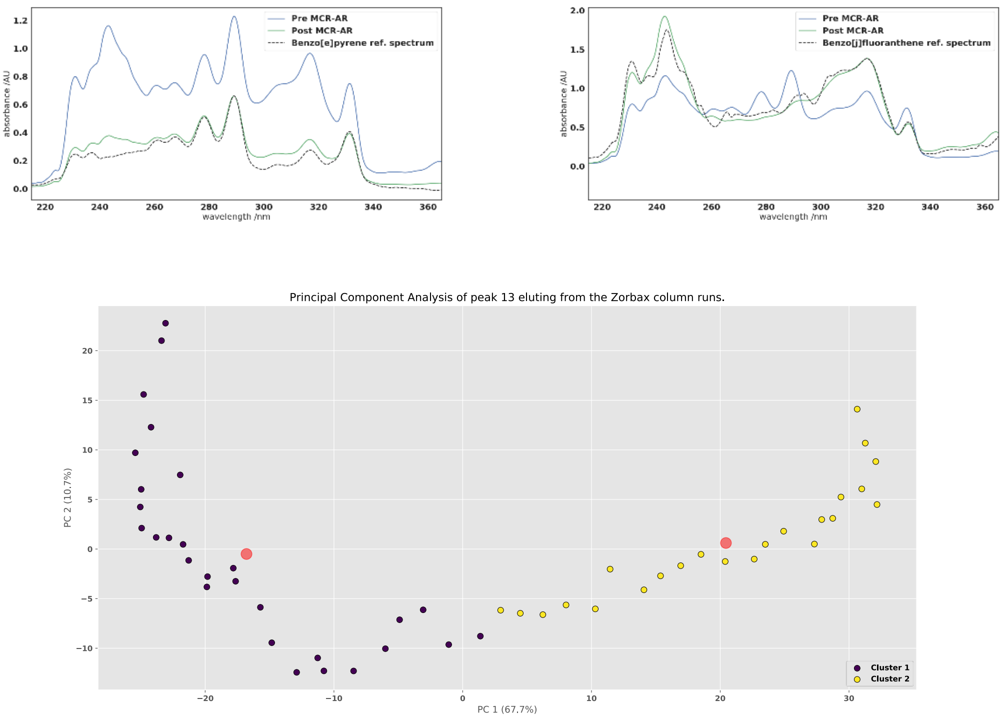

Supplementary Table 4 Literature reference spectra for spectral fingerprinting

Table containing all reference spectra sourced from literature sources.

Compound	Reference Spectra Literature Source
Naphthalene	1, 2, 3
Acenapthylene	2, 3, 4, 5
Acenapthene	1, 2, 5
Fluorene	2, 3, 5
Phenanthrene	1, 2, 3, 6
Anthracene	2, 3, 5, 6, 7
Fluoranthene	2, 3, 5, 6
Pyrene	1, 2, 5, 6
Benzo[c]phenanthrene	3
Chrysene	2, 3, 5, 8
Benzo[a]anthracene	2, 5, 8, 9
Benzo[j]fluoranthene	No literature spectra sourced
Benzo[e]pyrene	6, 3
Benzo[b]fluoranthene	2, 3, 5
Benzo[k]fluoranthene	2, 5, 7
Benzo[a]pyrene	2, 5, 6, 7
7-12-dimethylbenz[a]anthracene	No literature spectra sourced
Dibenz[a,h]anthracene	2, 3, 5
Benzo[ghi]perylene	2, 3, 5, 7
Indenol-cd-pyrene	2, 5
3-methyl-cholantrene	3
Dibenzo[a,l]pyrene	3
Dibenzo[a,h]pyrene	10, 11
Dibenzo[a,i]pyrene	No literature spectra sourced

Supplementary Figure 6 Classifier matched spectra (Poroshell column runs)

Classifier matched spectra for peaks eluting from Poroshell column runs on the *anywhereHPLC* portable instrument. Black line corresponds to the recorded spectrum from the chromatographic run. The red dashed line corresponds to the spectrum from our database that matched best to the PAH of interest. Peaks 2, 8, 11, 16 and 19 have multiple spectra since elution peaks contain multiple species; each component spectra are the result of spectral deconvolution of the peak.

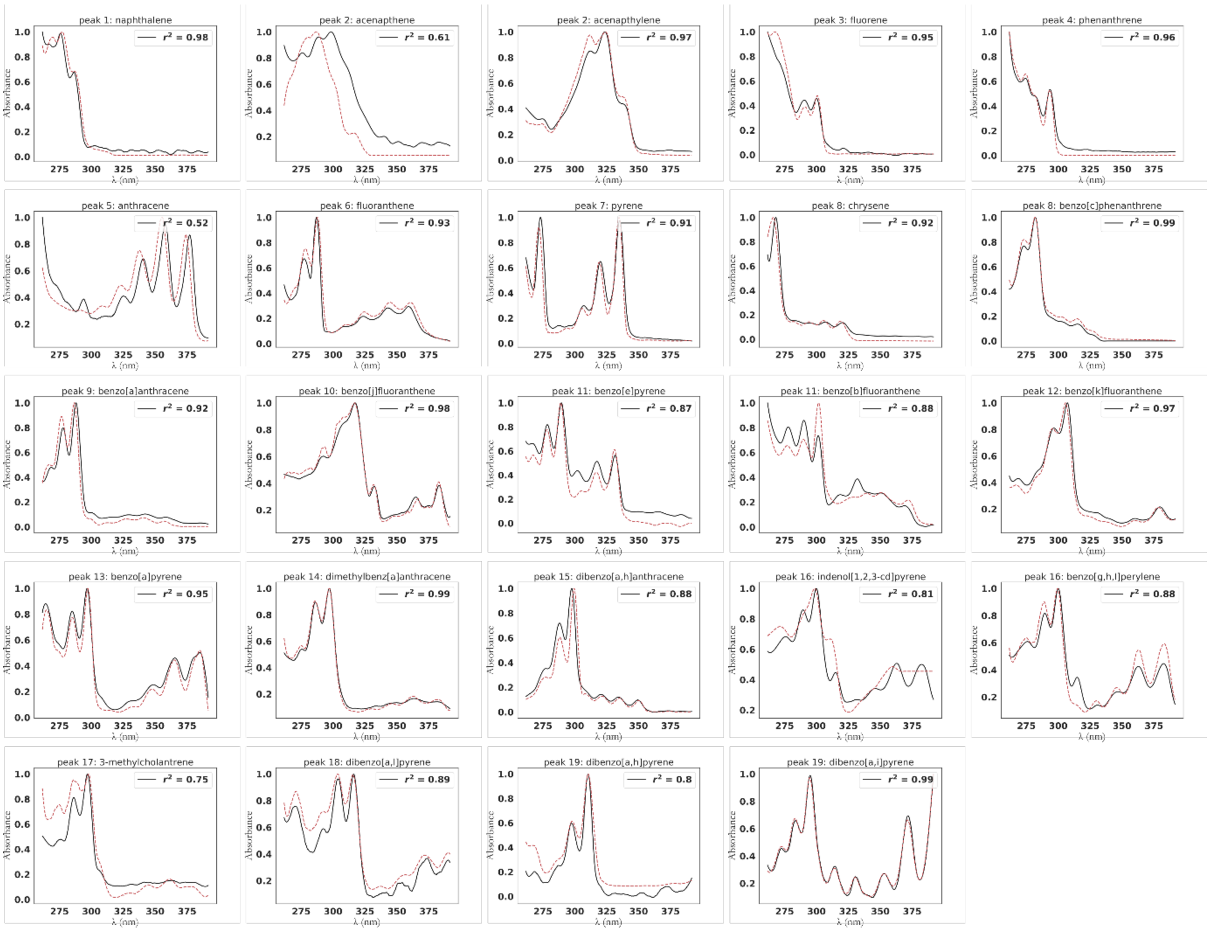

Supplementary Figure 7 Classifier matched spectra (Zorbax column runs)

Classifier matched spectra for peaks eluting from Zorbax column runs on the *anywhereHPLC* portable instrument. Black line corresponds to the recorded spectrum from the chromatographic run. The red dashed line corresponds to the spectrum from our database that matched best to the PAH of interest. Peaks 1, 12 and 21 have multiple spectra since elution peaks contain multiple species; each component spectra are the result of spectral deconvolution of the peak.

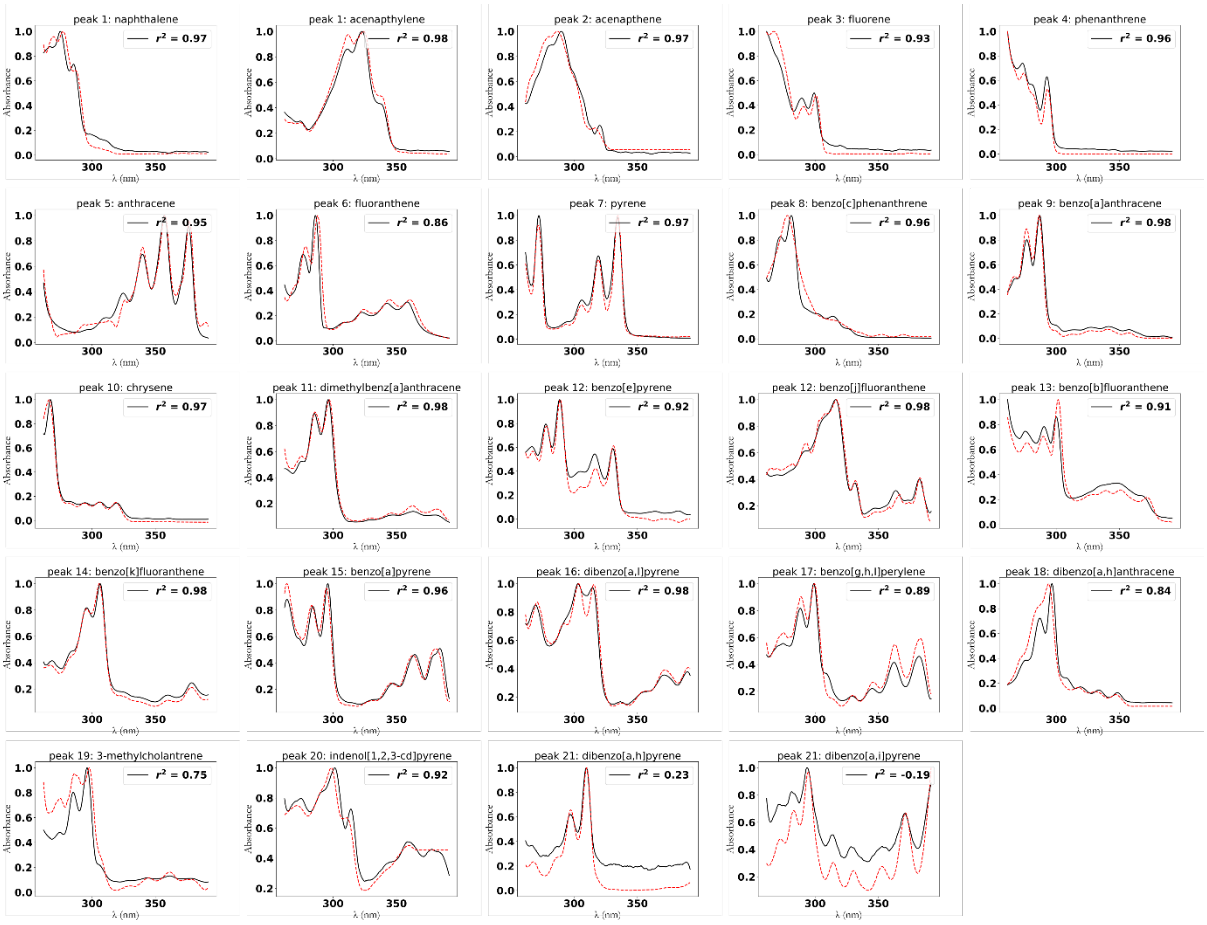

Supplementary Table 5 Limits of detection for standalone PAH species

Limits of detection for each PAH species determined using chemical standards run on the *anywhereHPLC* portable instrument using the Poroshell column. LODs were calculated at 3σ (standard deviation) variation in baseline noise above baseline average. LODs reported correspond to detection wavelength windows optimised for each individual PAH ($\lambda_{detection}$).

PAH species	LOD (ng/mL)	λ (nm)
naphthalene	46.27 ± 3.08	229.5 - 276.5
acenapthene	17.58 ± 0.85	229.5 - 233.8
acenapthylene	22.03 ± 1.14	229.5 - 244.5
fluorene	20.62 ± 0.28	229.5 - 276.5
phenanthrene	8.33 ± 0.15	229.5 - 265.9
anthracene	2.53 ± 0.06	229.5 - 255.2
fluoranthene	13.32 ± 0.35	229.5 - 255.2
pyrene	10.69 ± 0.56	229.5 - 265.9
benzo[c]phenanthrene	8.86 ± 1.21	229.5 - 287.1
chrysene	5.59 ± 0.010	229.5 - 276.5
benzo[a]anthracene	6.55 ± 0.090	229.5 - 297.7
benzo[j]fluoranthene	10.51 ± 0.82	229.5 - 244.5
benzo[e]pyrene	16.69 ± 1.54	229.5 - 287.1
benzo[b]fluoranthene	6.32 ± 0.10	229.5 - 265.9
benzo[k]fluoranthene	5.86 ± 0.08	229.5 - 255.2
benzo[a]pyrene	14.08 ± 0.52	229.5 - 265.9
7-12-dimethylbenz[a]anthracene	14.86 ± 1.19	229.5 - 244.5
dibenzo[a,h]anthracene	30.79 ± 1.90	229.5 - 308.3
benzo[ghi]perylene	55.80 ± 2.11	229.5 - 318.8
indenol[1,2,3-cd]pyrene	6.74 ± 0.33	229.5 - 255.2
3-methylcholantrene	7.80 ± 1.41	229.5 - 265.9
dibenzo[a,l]pyrene	6.80 ± 1.24	229.5 - 308.3
dibenzo[a,h]pyrene	25.34 ± 0.80	229.5 - 318.8
dibenzo[a,i]pyrene	40.75 ± 0.72	229.5 - 244.5

Supplementary Table 6 Field sample results – spike PAH recovery rate

Recoveries rates of PAHs measured using spiked samples into water sources in the field.

**PAH RECOVERY RATE**				**Field Site**		
**Species**	**W**	**C**	**L1**	**L2**	**L3**	**L4**
naphthalene	119.7%	88.9%	104.2%	98.5%	95.7%	116.3%
acenapthene	99.3%	82.2%	114.9%	97.8%	109.1%	116.3%
acenapthylene	98.3%	94.3%	114.8%	98.0%	107.0%	116.2%
fluorene	109.6%	99.6%	96.9%	99.8%	94.2%	98.6%
phenanthrene	113.3%	93.1%	104.0%	100.1%	98.6%	105.7%
anthracene	117.4%	95.6%	100.6%	100.1%	101.5%	106.7%
fluoranthene	115.8%	94.8%	104.0%	100.0%	94.8%	104.3%
pyrene	117.1%	94.9%	101.6%	100.1%	97.9%	104.0%
benzo[c]phenanthrene	102.0%	87.6%	103.3%	100.3%	100.3%	103.5%
chrysene	102.5%	86.9%	103.3%	100.2%	101.1%	103.6%
benzo[a]anthracene	117.5%	94.0%	103.1%	100.0%	97.5%	104.1%
benzo[j]fluoranthene	120.7%	88.1%	106.0%	100.2%	98.9%	109.1%
benzo[e]pyrene	110.8%	90.6%	112.9%	100.0%	104.8%	111.9%
benzo[b]fluoranthene	110.1%	85.8%	110.5%	99.1%	103.9%	111.1%
benzo[k]fluoranthene	117.9%	91.3%	103.8%	98.2%	98.0%	107.8%
benzo[a]pyrene	117.7%	90.1%	104.2%	98.0%	94.8%	106.1%
7-12-dimethylbenz[a]anthracene	90.9%			98.1%		
dibenzo[a,h]anthracene	125.5%	95.5%	104.3%	98.5%	100.0%	110.3%
benzo[ghi]perylene	91.1%	91.2%	105.2%	97.8%	102.1%	103.0%
indenol[1,2,3-cd]pyrene	92.1%	92.2%	106.3%	98.9%	103.2%	104.1%
3-methylcholantrene	138.5%	89.6%	104.9%	115.9%	98.4%	104.6%
dibenzo[a,l]pyrene	170.4%	109.0%	102.0%	116.5%	119.6%	102.7%
dibenzo[a,h]pyrene	102.7%	85.2%	98.3%	106.2%	98.7%	104.3%
dibenzo[a,i]pyrene	110.9%	85.9%	103.3%	110.3%	105.9%	106.9%

Supplementary Table 7 Field sample results – variation in spike PAH recovery rate

Variation of recoveries rates of PAHs measured using spiked samples into water sources in the field. Values are standard deviations of PAH recovery rate (n = 3).

**VARIATION IN PAH RECOVERY RATE**				**Field Site**		
**Species**	**W**	**C**	**L1**	**L2**	**L3**	**L4**
naphthalene	1.0%	1.3%	3.7%	1.9%	3.7%	2.5%
acenapthene	6.6%	13.7%	1.4%	7.6%	4.9%	1.9%
acenapthylene	7.2%	5.4%	2.5%	12.1%	1.7%	2.4%
fluorene	2.3%	1.9%	1.5%	0.7%	0.4%	1.1%
phenanthrene	1.1%	6.2%	2.0%	0.3%	0.7%	2.0%
anthracene	1.0%	5.3%	10.7%	1.0%	0.9%	3.0%
fluoranthene	2.0%	4.1%	2.0%	1.9%	2.9%	3.1%
pyrene	2.5%	4.8%	2.3%	0.3%	1.1%	2.3%
benzo[c]phenanthrene	0.1%	4.9%	1.0%	1.0%	0.1%	0.9%
chrysene	0.5%	4.8%	0.9%	0.7%	0.1%	0.6%
benzo[a]anthracene	2.7%	5.0%	2.7%	1.9%	1.1%	2.9%
benzo[j]fluoranthene	2.6%	6.0%	2.9%	0.8%	1.7%	3.4%
benzo[e]pyrene	0.0%	5.6%	2.4%	2.4%	1.3%	2.6%
benzo[b]fluoranthene	0.4%	4.8%	2.1%	2.0%	1.2%	2.6%
benzo[k]fluoranthene	2.2%	4.4%	2.4%	1.5%	2.0%	3.1%
benzo[a]pyrene	1.0%	12.6%	3.4%	3.1%	1.8%	1.6%
7-12-dimethylbenz[a]anthracene	38.4%			23.5%		
dibenzo[a,h]anthracene	1.7%	18.0%	3.8%	2.9%	2.7%	4.9%
benzo[ghi]perylene	8.4%	6.6%	3.0%	9.8%	5.1%	3.2%
indenol[1,2,3-cd]pyrene	8.4%	6.6%	3.0%	9.8%	5.1%	3.2%
3-methylcholantrene	4.3%	17.7%	5.5%	3.3%	2.4%	7.5%
dibenzo[a,l]pyrene	10.9%	33.4%	9.6%	3.2%	4.3%	10.1%
dibenzo[a,h]pyrene	1.7%	8.2%	4.5%	5.5%	1.2%	4.5%
dibenzo[a,i]pyrene	0.3%	7.2%	3.3%	3.3%	2.0%	4.0%

Supplementary Figure 8 Field sample results – sample quality

Field samples tested and collected at sites W (Wales), Cyprus (C) and 4 locations in London: the River Pinn (L1), Paddington arm of the Grand Union Canal (L2), Millwall Dock on the River Thames (L3) and rainwater collected in North-West Greater London (L4). See main text for location details. a) Photograph of samples collected and stored in 50 mL tubes showing turbidity caused by suspended solids and organic matter. b) Samples were tested for 14 measures of water quality (see colour key) using commonly available testing strips (Bebapanda, China). Samples are tested in triplicate with a comparison to HPLC grade water from the lab.

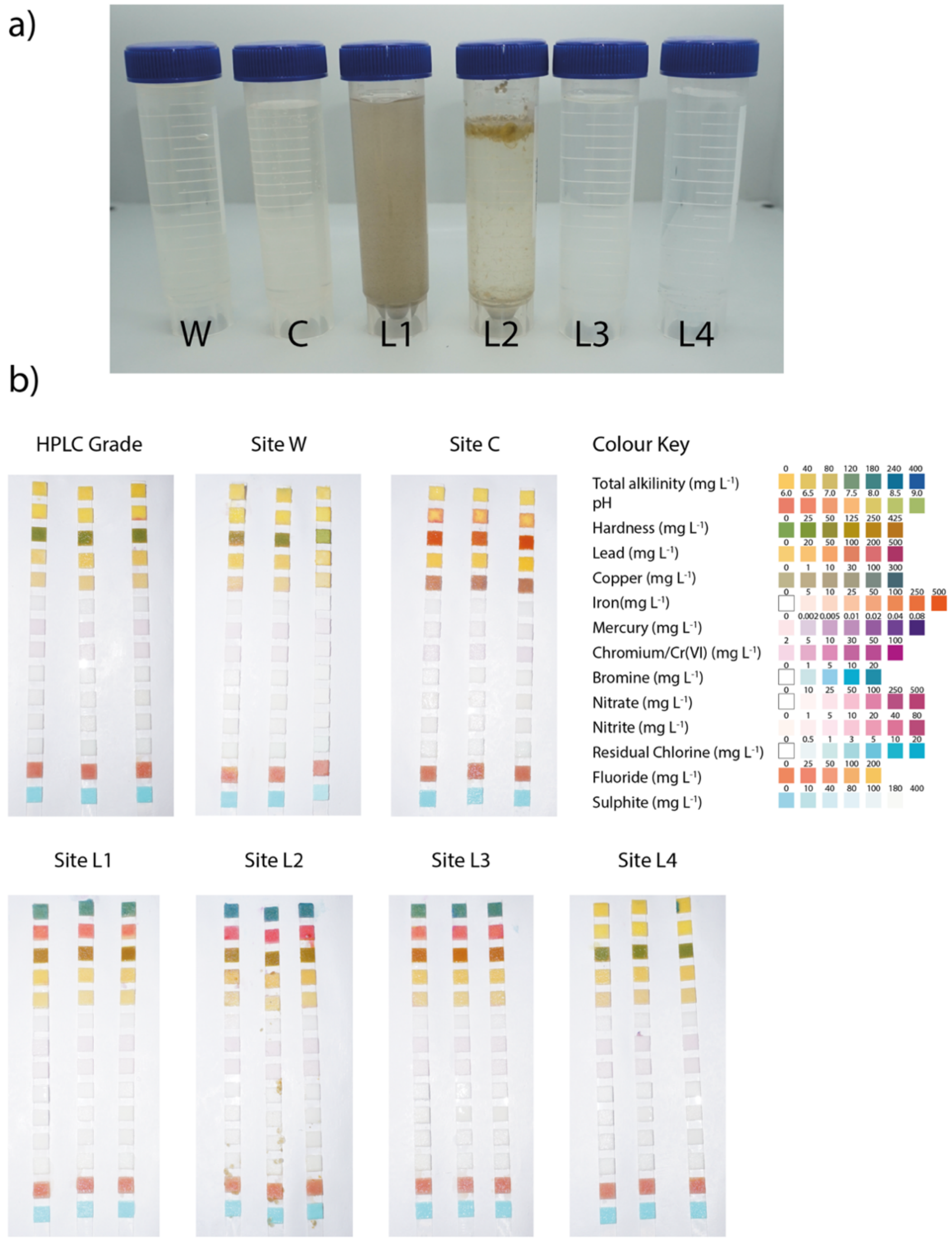

Supplementary Table 8 Field sample results – sample quality

Interpretation of water quality test strip (**Supplementary Figure 8b**) results for each sample. Ranges are provided for results that either span values or are difficult to interpret.

	**Field Site**					
**Test**	**W**	**C**	**L1**	**L2**	**L3**	**L4**
Total alkalinity (mg L^{-1})	40	0-40	120-180	240	180	0
pH	8.0	6.5-7.5	6.5-7.0	6.0	6.0-6.5	8.0
Hardness (mg L^{-1})	25-50	425	250-425	125	250-425	25-50
Lead (mg L^{-1})	0	0	0	0	0	0
Copper (mg L^{-1})	0	100	0	0	0	0
Iron (mg L^{-1})	0	0	0	0	0	0
Mercury (mg L^{-1})	0	0	0	0	0.002	0-0.002
Chromium/Cr(VI) (mg L^{-1})	2	2-5	2	2	2	2
Bromine (mg L^{-1})	0	0	0	0	0	0
Nitrate (mg L^{-1})	0	0	0	0	0	0
Nitrite (mg L^{-1})	0	0	0	0	0	0
Residual Chlorine (mg L^{-1})	0	0	0	0	0	0
Fluoride (mg L^{-1})	50	0-25	0-50	0-50	0-50	0-50
Sulphite (mg L^{-1})	10	0	0	0	0	0-10

Supplementary Figure 9 Evaluation of portable instrument using the BETTER criteria.

The BETTER (portaBle fiEld Testing sTandard framEwoRk) criteria 2020 act as a framework to facilitate more objective comparisons, encourage better reporting, and act as a development framework for the portable HPLC community.[13] BETTER grade levels (each ranging from 1 to 5) are selected both to represent the current range of developments, and community goals. To date, portable LC systems meet some of the grade 2 and 3 criteria. The grade 4 and 5 criteria are deemed a challenge to be met by next generation devices; indeed, no instrument reported to date reaches Grade 5 in any category. A public GitHub repository of the current BETTER standards, and notes on how to perform assessments, are maintained at https://better-hplc.github.io.

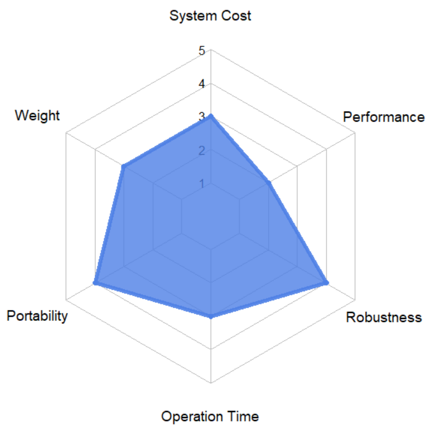

System Cost	Weight	Portability	Operation Time	Robustness	Performance
3	3	4	3	4	2

Supplementary References

1. Halasinski, T. M., Salama, F. & Allamandola, L. J. Investigation of the Ultraviolet, Visible, and Near-Infrared Absorption Spectra of Hydrogenated Polycyclic Aromatic Hydrocarbons and Their Cations. *Astrophys. J.* **628**, 555–566 (2005).
2. Tropp, J. *et al.* A sensor array for the discrimination of polycyclic aromatic hydrocarbons using conjugated polymers and the inner filter effect. *Chem. Sci.* **10**, 10247–10255 (2019).
3. Talrose, V. *et al. UV/Visible Spectra in NIST Chemistry WebBook*. (2020). doi: 10.18434/T4D303
4. Thomas, O. & Causse, J. *UV-Visible Spectrophotometry of Water and Wastewater*. (Elsevier, 2017). doi: 10.1016/B978-0-444-63897-7.00002-0
5. Joseph, M. Identification of Polynuclear Aromatic Hydrocarbons in a Complex Matrix with Diode Array Detection., **5**, 13–16, https://www.agilent.com/cs/library/applications/lc08.pdf, (2020).
6. Climate Policy Watcher - Phenanthrene Uv-Vis., *Climate Policy Watcher*, https://www.climate-policy-watcher.org/aromatic-hydrocarbons/info-lei.html, (2020).
7. Prakash, J. & Mishra, A. K. Simultaneous Quantification of Multiple Polycyclic Aromatic Hydrocarbons in Aqueous Media using Micelle Assisted White Light Excitation Fluorescence. *Sci. Rep.* **10**, 1–10 (2020).
8. Lopes, W. A., Afonso, P., Pereira, D. P. & Viertler, H. and 3-Nitrofluoranthene and their Correlation with Direct-Acting Mutagenicities. *Potentials* **16**, 1099–1103 (2005).
9. Rivera-Figueroa, A. M., Ramazan, K. A. & Finlayson-Pitts, B. J. Fluorescence, Absorption, and Excitation Spectra of Polycyclic Aromatic Hydrocarbons as a Tool for Quantitative Analysis. *J. Chem. Educ.* **81**, 242–245 (2004).
10. Wang, H., Szczepanski, J., Hirata, S. & Vala, M. Vibrational and electronic absorption spectroscopy of dibenzo[b,def] chrysene and its ions. *J. Phys. Chem. A* **109**, 9737–9746 (2005).
11. Alcanzare, R. J. C. Polycyclic aromatic compounds in wood soot extracts from Henan, China., https://digitalcommons.lsu.edu/gradschool_theses/2377, (2006).
12. Erokhin, K. S., Gordeev, E. G. & Ananikov, V. P. Revealing interactions of layered polymeric materials at solid-liquid interface for building solvent compatibility charts for 3D printing applications. *Sci. Rep.* **9**, 20177 (2019).
13. Rahimi, F. *et al.* A Review of Portable High-Performance Liquid Chromatography: The Future of the Field? *Chromatographia* (2020). doi:10.1007/s10337-020-03944-6
